# Global, regional, and national burden of forearm fracture from 1990 to 2021: a study based on GBD 2021

**DOI:** 10.3389/fpubh.2025.1638277

**Published:** 2025-07-25

**Authors:** Cheng Chen, ShuTao Zhang, ZhenDong Li, HaiChao Zhou, Jiang Xia, Bing Li, YunFeng Yang

**Affiliations:** ^1^Department of Orthopedics, Tongji Hospital, School of Medicine, Tongji University, Shanghai, China; ^2^Department of Orthopedics, Shanghai Sixth People's Hospital Affiliated to Shanghai Jiao Tong University School of Medicine, Shanghai, China; ^3^Department of Orthopaedics, Ruijin Hospital, Shanghai JiaoTong University School of Medicine, Shanghai, China

**Keywords:** epidemiology, incidence, YLDs, GBD 2021, forearm fracture

## Abstract

**Objective:**

To comprehensively examine the regional, national, and global burden and trends of forearm fractures from 1990 to 2021.

**Methods:**

The incidence and years lived with disability (YLDs), with uncertainty interval (UI), for forearm fractures from 1990 to 2021 were extracted from the Global Burden of Disease 2021. The temporal, geographical, and demographic burden of forearm fractures was assessed, as well as the leading causes. Finally, a decomposition analysis was performed.

**Results:**

In 1990, the incidence number of forearm fractures globally was 26,098,810 (95% UI: 20,967,988–32,372,267), which increased to 31,905,396 (95% UI: 25,403,829–39,982,115) in 2021. The age-standardized incidence rate was 483.28 (95% UI: 387.42–599.37) in 1990 and decreased to 402.35 (95% UI: 319.86–505.21) in 2021. In 1990, the global number of YLDs due to forearm fractures was 144,166 (95% UI: 87,129–229,017), which increased to 205,031 (95% UI: 126,061–320,235) in 2021. The age-standardized YLDs rate was 2.98 (95% UI: 1.82–4.7) in 1990, which decreased to 2.51 (95% UI: 1.54–3.93) in 2021. Females showed a higher burden than males, and the incidence rate of forearm fractures exhibited a bimodal distribution, peaking in youth and older adulthood. Recently, the disease burden presented a challenge due to the aging population. Falls were the most prominent cause of forearm fractures, followed by road injuries and exposure to mechanical forces.

**Conclusion:**

Forearm fractures have increased in incidence and YLDs number since 1990, particularly among females. The incidence rate follows a bimodal distribution, with peaks in youth and older adulthood. The age-related burden has progressively shifted toward older populations, reflecting trends in global aging. Falls remain the leading cause of forearm fractures, with the highest burden observed among older adult females.

## 1 Introduction

Forearm fractures are among the most common types of fractures worldwide. According to the Global Burden of Disease (GBD) 2019 study, the global incidence rate of forearm fractures in 2019 was estimated at 396.6 per 100,000, second only to fractures of the patella, tibia, fibula, or ankle, with a corresponding years lived with disability (YLDs) rate of 2.7 (1). Forearm fractures can result from direct or, more commonly, indirect trauma, typically resulting from falls (2). Osteoporosis is a significant risk factor (3).

The functional importance of the forearm is substantial, having a pivotal role in activities of daily living, occupational tasks, and recreational pursuits. Fractures can result in significant disability and decreased quality of life. The biomechanical and anatomical complexity of the forearm includes its joint structure and intricate musculature and tendons; together, these features add unique challenges to treatment and rehabilitation. Therefore, a clear understanding of the epidemiology of forearm fractures is essential to guide prevention efforts, optimize management strategies, and minimize their impact on individuals and society.

Current treatment focuses on restoring anatomical alignment and functional capacity. Surgical intervention may be necessary for unstable or displaced fractures when closed reduction is insufficient. With the global population aging, fracture incidence is increasing, particularly in the older adult. Forearm fractures in this group not only affect quality of life but also increase the burden on the medical system. The financial costs associated with treating forearm fractures impose a substantial economic burden on healthcare systems (4, 5).

Despite their prevalence, global epidemiological studies have not clearly identified the burden and trends of forearm fractures (6–10). Prior literature using GBD 2019 data lacked depth and did not incorporate the latest 2021 estimates (1). Little is known about how the burden varies across geographic regions, age, and gender. Given the significant health and socioeconomic impact, an updated, comprehensive analysis is needed to inform targeted interventions and allocate healthcare resources. Therefore, in the current study, we aimed to comprehensively examine the global, regional, and national burden and trends of forearm fractures from 1990 to 2021 using data from the GBD 2021.

## 2 Methods

The GBD study is a global health research initiative that offers a unique perspective on understanding trends and inequalities. GBD 2021 is the latest iteration of this ongoing effort, representing the forefront of global health epidemiology. It compiles evidence from multiple global data sources, representing a robust tool aimed at understanding the burden of diseases and injuries and informing public health interventions, ultimately contributing to evidence-based decision-making (11–13). The GBD 2021 study utilizes a wide range of input data, including demographic information, disease registries, and survey data, which undergo rigorous methodological processing to generate comparable estimates. The GBD encompasses 204 countries and territories, 371 diseases and injuries, and 88 risk factors, presenting a comprehensive landscape of global health.

We extracted data on the incidence and YLDs of radius and/or ulna fractures from 1990 to 2021 using the GBD Results tool.^1^ The 95% uncertainty interval (UI) represented the 2.5th and 97.5th percentiles of the sampling distribution. The GBD 2021 established 21 GBD regions, each comprising multiple countries or territories with comparable characteristics, allowing focused research and policy formulation to address specific regional health challenges. We conducted a systematic assessment of the geographical, demographic, and temporal burden of forearm fractures. To quantify the trends between 1990 and 2021, the estimated annual percentage change (EAPC) was calculated with a 95% confidence interval (CI) (14); a positive EAPC indicates an increasing trajectory, while a negative EAPC denotes a decreasing trend. Additionally, we analyzed the leading causes of forearm fractures. Finally, the Das Gupta decomposition analysis was performed (15), which partitions the total variation into three distinct components: alterations in disease epidemiology, population change, and aging. Decomposition analysis assesses the independent impacts of each factor on the overall alterations in the disease burden. All statistical analyses and data visualization were conducted in R software (version 4.2.2).

## 3 Results

### 3.1 Burden of forearm fractures: global trends

**Figure 1:**
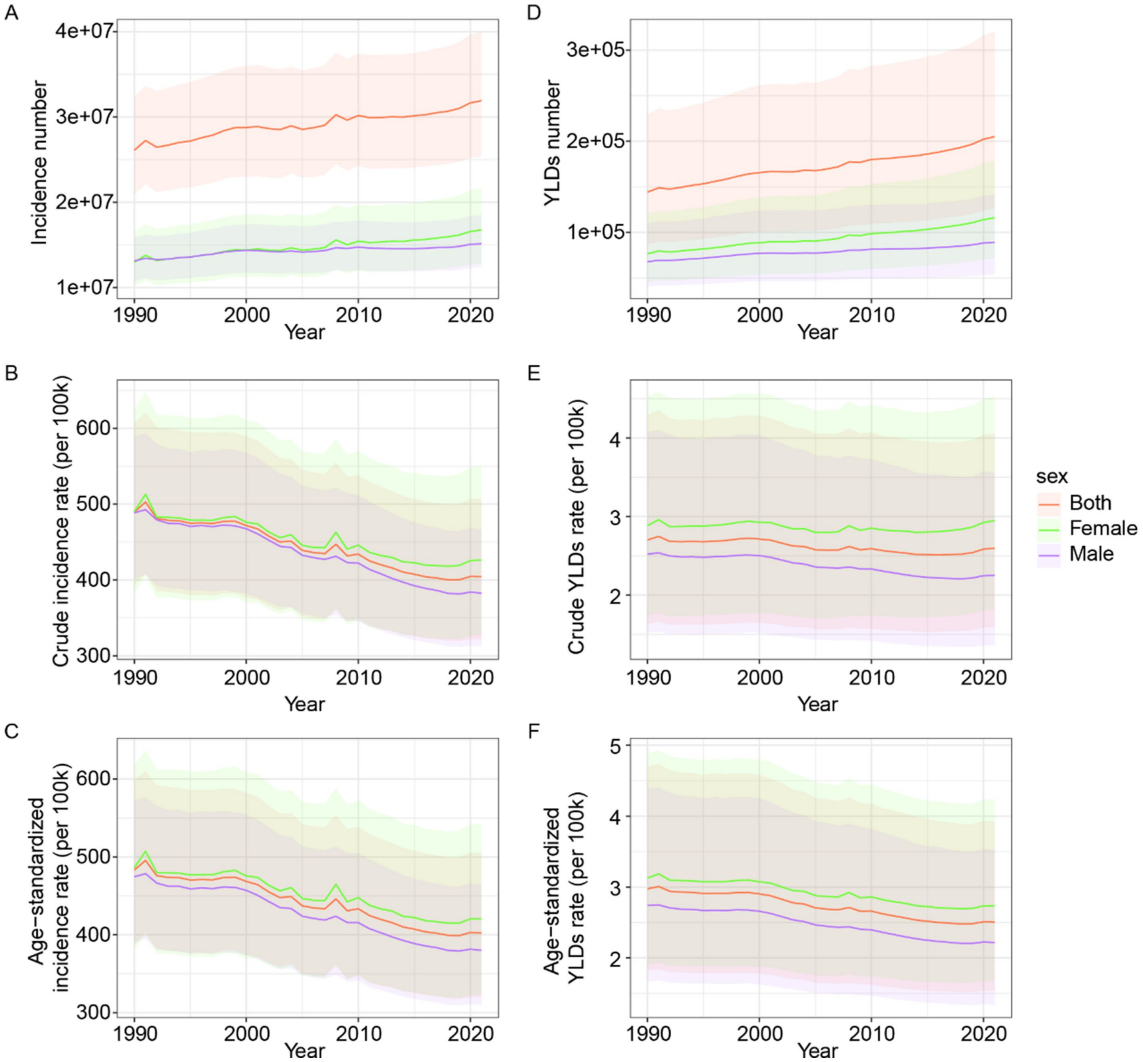
Global epidemiological trends of forearm fracture across gender from 1990 to 2021. **(A)** Incidence number. **(B)** Crude incidence rate. **(C)** Age-standardized incidence rate. **(D)** YLDs number. **(E)** Crude YLDs rate. **(F)** Age-standardized YLDs rate. YLDs, years lived with disability.

From 1990 to 2021, the global incidence rate of forearm fractures exhibited a decreasing trend, while YLDs rate remained relatively stable. However, the absolute number of incidence and YLDs still showed an increasing trend (Figure 1). Females bore a higher burden than males (Figure 1).

In 1990, the global incidence number of forearm fractures was 26,098,810 (95% UI: 20,967,988–32,372,267), which increased to 31,905,396 (95% UI: 25,403,829–39,982,115) in 2021. The crude incidence rate of forearm fractures globally was 489.33 (95% UI: 393.13–606.95) in 1990 and decreased to 404.31 (95% UI: 321.92–506.66) in 2021. The age-standardized incidence rate was 483.28 (95% UI: 387.42–599.37) in 1990, which decreased to 402.35 (95% UI: 319.86–505.21) in 2021.

In 1990, the global number of YLDs was 144,166 (95% UI: 87,129–229,017), which increased to 205,031 (95% UI: 126,061–320,235) in 2021. The crude YLDs rate was 2.7 (95% UI: 1.63–4.29) in 1990 and slightly decreased to 2.6 (95% UI: 1.6–4.06) in 2021. Finally, the age-standardized YLDs rate was 2.98 (95% UI: 1.82–4.7) in 1990, which decreased to 2.51 (95% UI: 1.54–3.93) in 2021.

### 3.2 Burden of forearm fractures: regional trends

The GBD regions with the highest EAPC of age-standardized incidence rates were the Caribbean [0.35 (95% CI: −0.35–1.06)], Oceania [0.27 (95% CI: −0.15–0.7)], and Southern Latin America (0.14 (95% CI: −0.04 to 0.33)). In 2021, the GBD regions with the highest age-standardized incidence rates were Eastern Europe [943.29 (95% UI: 755.11–1,173.17)], Central Europe [909.72 (95% UI: 720.21–1,119.3)], and Australasia [594.58 (95% UI: 428.55–792.88)].

**Figure 2:**
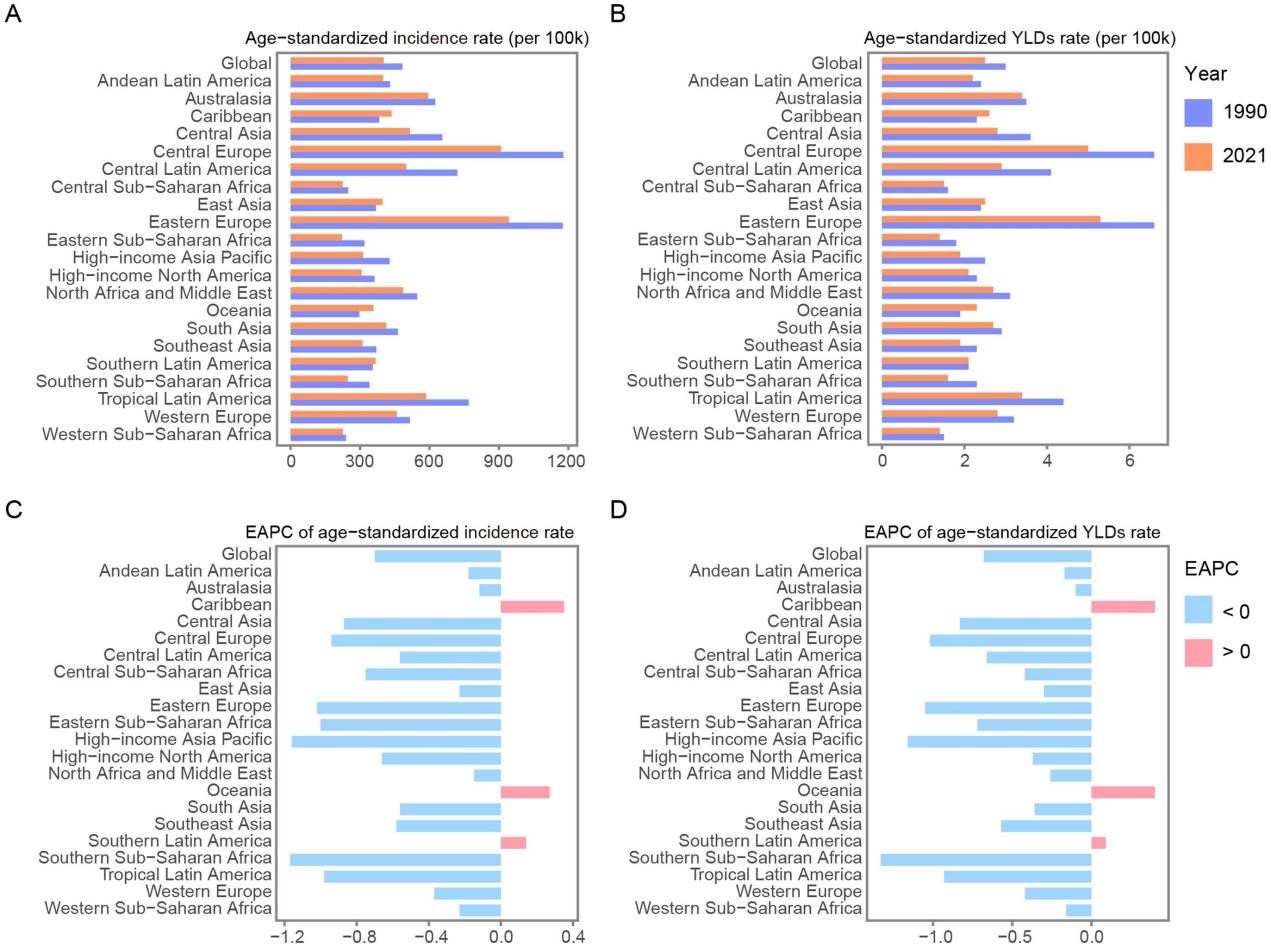
Age-standardized incidence **(A)** and YLDs rate **(B)** of forearm fracture in GBD regions and globe in 1990 and 2021. EPAC of age-standardized incidence **(C)** and YLDs rate **(D)** of forearm fracture in GBD regions and globe from 1990 to 2021. YLDs, years lived with disability; EAPC, estimated annual percentage change.

The GBD regions with the highest EAPC of age-standardized YLDs rates were Oceania [0.4 (95% CI: 0.16–0.65)], the Caribbean [0.4 (95% CI: −0.05–0.86)], and Southern Latin America [0.09 (95% CI: −0.07–0.25)]. In 2021, the GBD regions with the highest age-standardized YLDs rates were Eastern Europe [5.25 (95% UI: 3.12–8.3)], Central Europe [4.99 (95% UI: 2.99–8.05)], and Tropical Latin America [3.39 (95% UI: 2.03–5.25)]. Further details can be found in Figure 2 and Supplementary Tables S1, S2.

### 3.3 Burden of forearm fractures: national and territorial trends

The countries and territories with the highest EAPC of age-standardized incidence rates were the Syrian Arab Republic [2.61 (95% CI: 1.7–3.54)], Libya [1.08 (95% CI: 0.62–1.54)], and Yemen [0.89 (95% CI: 0.57–1.22)]. In 2021, the countries and territories with the highest age-standardized incidence rates were Slovenia [1,172.8 (95% UI: 916.72–1,469.73)], Saudi Arabia [1,068.92 (95% UI: 842.57–1,346.95)], and Slovakia [1,059.01 (95% UI: 825.72–1,314.3)].

**Figure 3:**
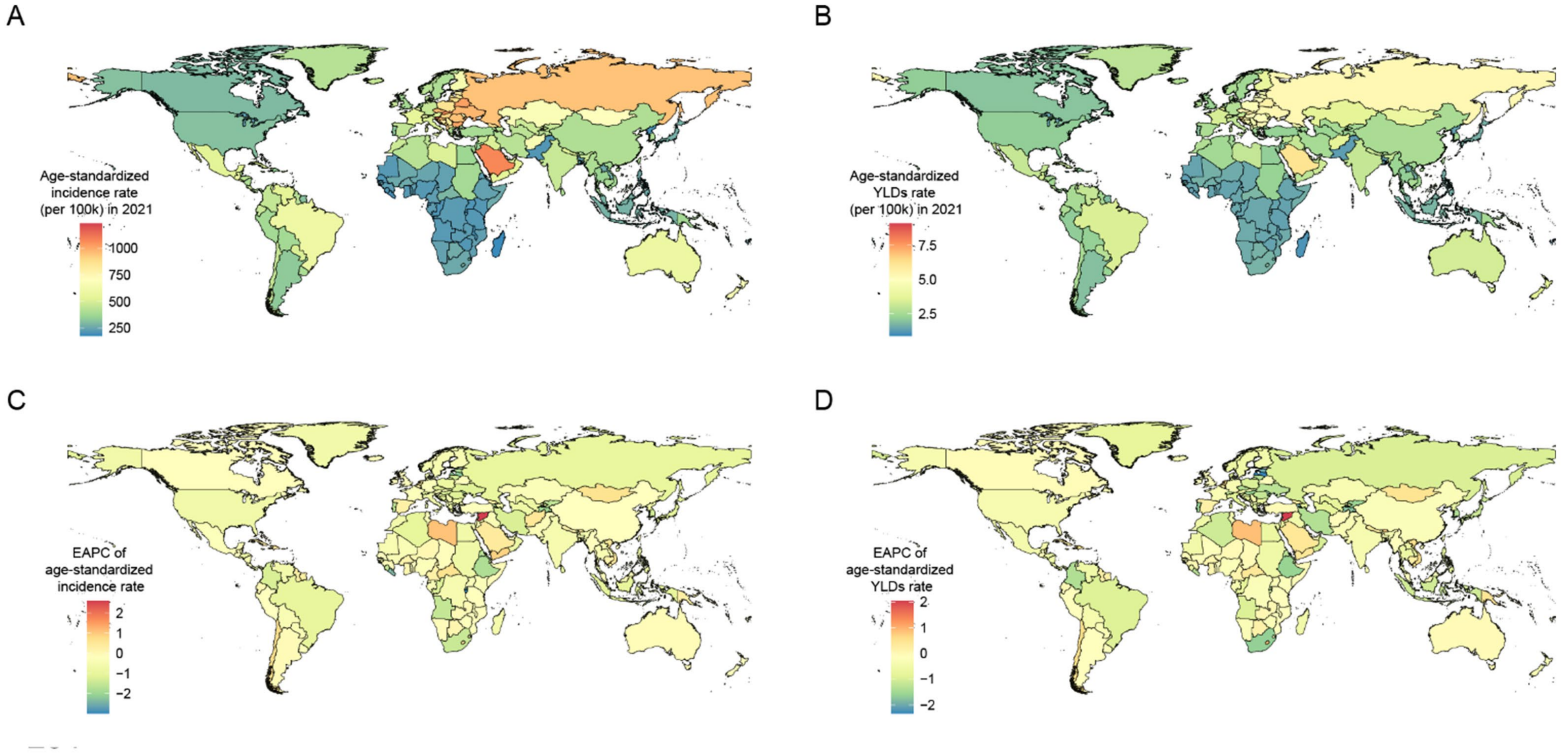
Age-standardized incidence rate **(A)** and age-standardized YLDs rate **(B)** maps of forearm fracture by countries and territories in 2021. EPAC maps of age-standardized incidence rate **(C)** and age-standardized YLDs rate **(D)** of forearm fracture by countries and territories from 1990 to 2021. YLDs, years lived with disability; EAPC, estimated annual percentage change.

The countries and territories with the highest EAPC of age-standardized YLDs rates were the Syrian Arab Republic [2.05 (95% CI: 1.46–2.64)], Libya [0.88 (95% CI: 0.56–1.21)], and Lesotho [0.87 (95% CI: 0.72–1.03)]. In 2021, the countries and territories with the highest age-standardized YLDs rates were Slovenia [6.57 (95% UI: 3.97–10.47)], Saudi Arabia [6.15 (95% UI: 3.74–9.57)], and Slovakia [5.84 (95% UI: 3.51–9.36)]. More details are presented in Figure 3 and Supplementary Tables S3, S4.

### 3.4 Burden of forearm fractures: age and gender trends

**Figure 4:**
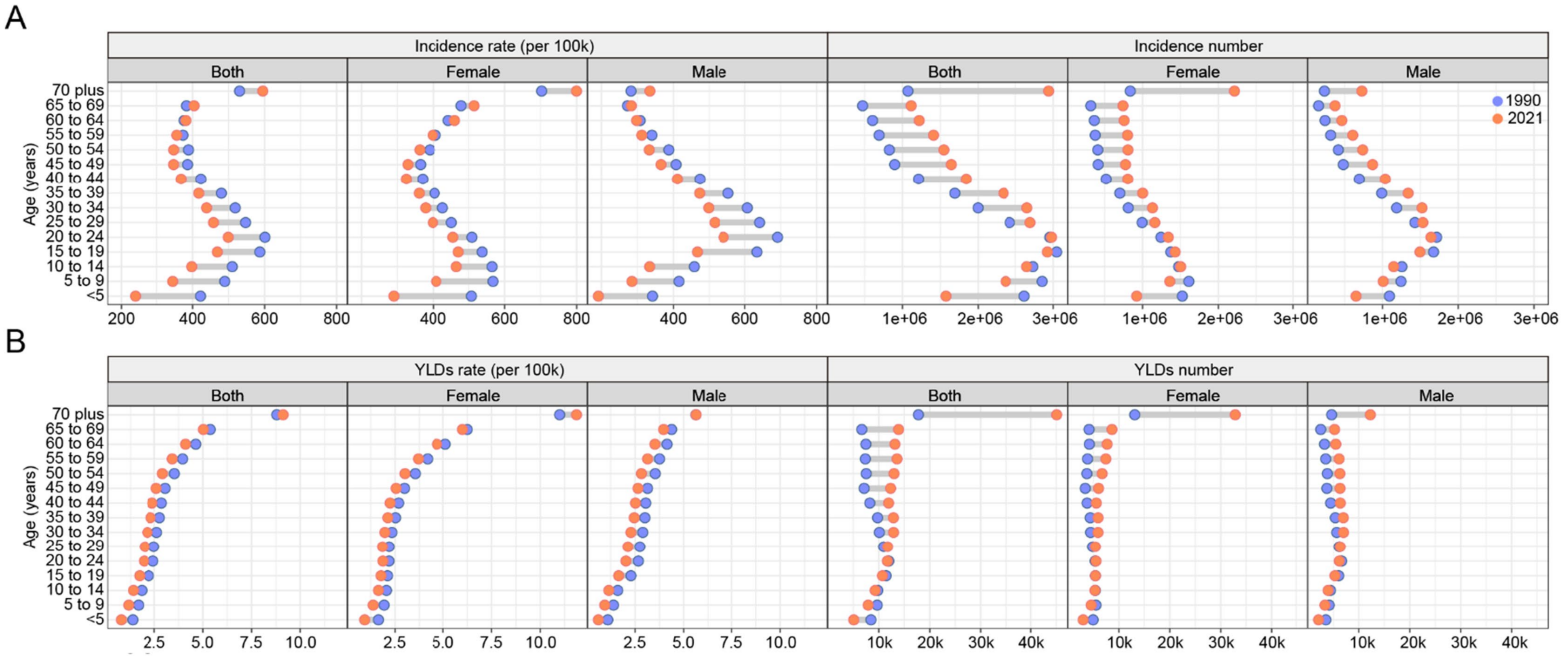
The change of global incidence **(A)** and YLDs **(B)** rate and number of forearm fracture across age and gender from 1990 to 2021. YLDs, years lived with disability.

The incidence rate of forearm fractures across all genders exhibits a bimodal distribution, with peaks occurring in young and older adult individuals. When examining the incidence rates specifically within female and male populations, a similar bimodal pattern emerges, though the first peak occurs earlier in females during adolescence, while for males, it occurs in young adulthood (Figure 4). The age distribution of YLDs rates for forearm fractures is similar between different gender groups, with rates generally increasing as age increases (Figure 4).

Trends in both incidence and YLDs rates from 1990 to 2021 indicate a decreasing burden over time for all groups except the older adult. However, when considering the incidence and YLDs number of forearm fracture, the burden has increased across all groups from 1990 to 2021, with the most significant rise observed in the older adult population (Figure 4).

### 3.5 Burden of forearm fractures: leading causes

**Figure 5:**
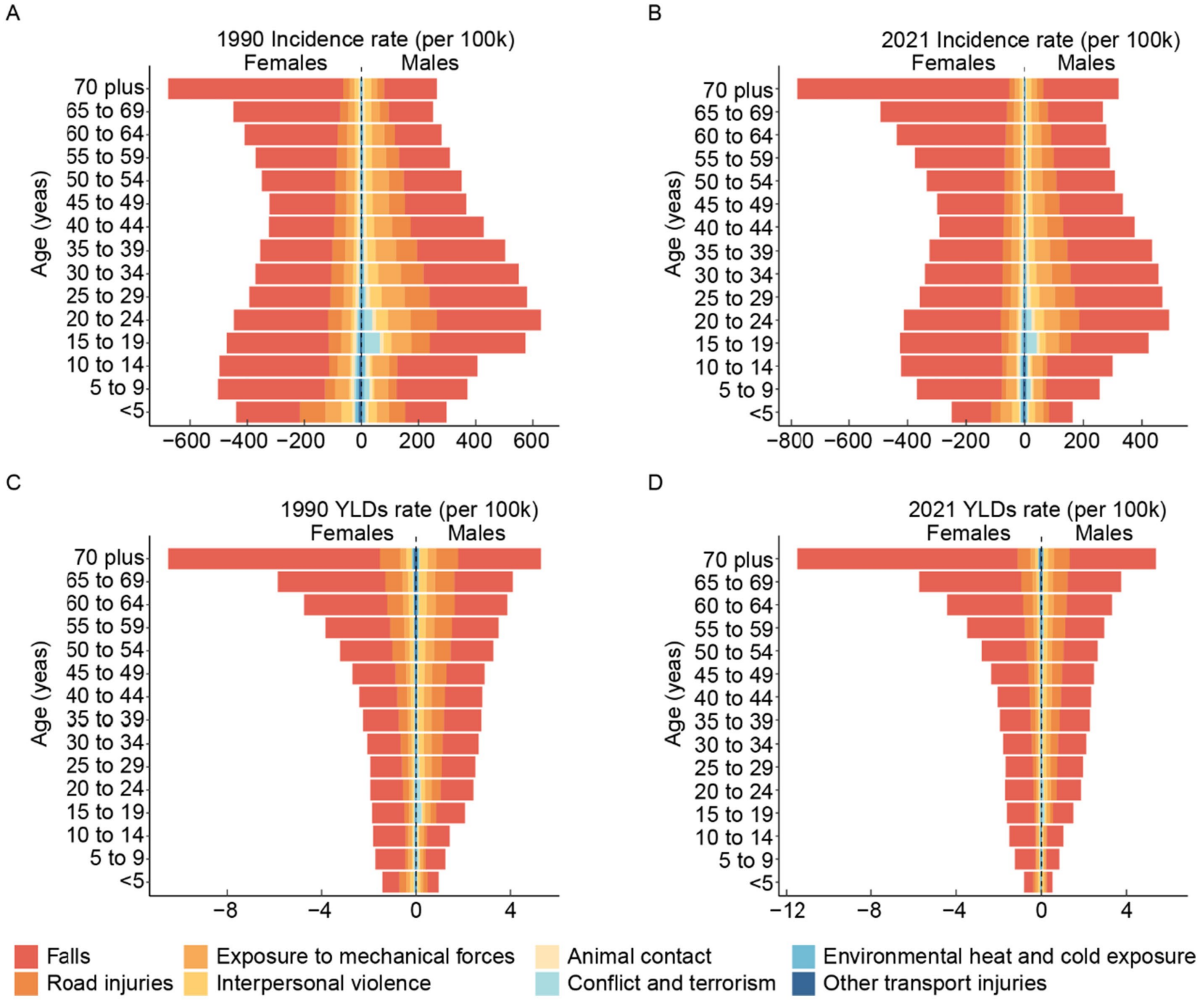
The leading causes of global incidence rates of forearm fracture across age and gender in 1990 **(A)** and 2021 **(B)**. The leading causes of global YLDs rates of forearm fracture across age and gender in 1990 **(C)** and 2021 **(D)**. YLDs, years lived with disability.

Falls were the most predominant cause of forearm fractures, followed by road injuries and exposure to mechanical forces (Figure 5). In 2021, the age-standardized and crude incidence rates for forearm fractures caused by falls were 269.61 (95% UI: 189.54–368.05) and 272.41 (95% UI: 191.91–371.89), respectively. Additionally, in 2021, the age-standardized YLDs rate for forearm fractures attributed to falls was 1.72 (95% UI: 1.04–2.65), while the crude YLDs rate was 1.78 (95% UI: 1.08–2.75). Notably, the disease burden was the most prominent among older adult women (Figure 5).

### 3.6 Burden of forearm fractures: decomposition analysis

**Figure 6:**
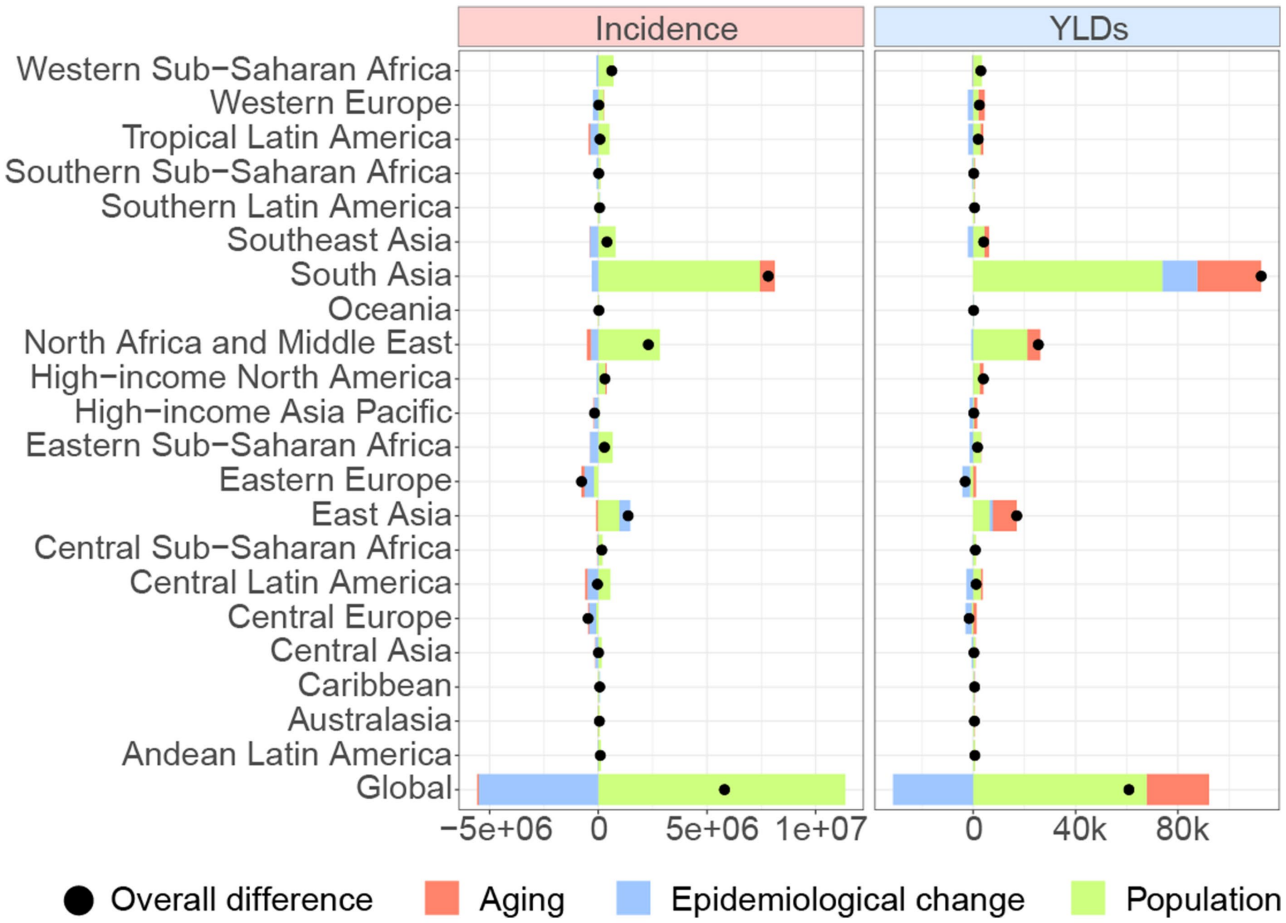
Changes in incidence and YLDs of forearm fracture according to population-level determinants including aging, population growth and epidemiological change from 1990 to 2021 at the global level and GBD regions. YLDs, years lived with disability.

From 1990 to 2021, the global incidence and YLDs significantly increased, with the highest growth observed in South Asia (Figure 6). Aging, population growth, and epidemiological changes contribute to the global incidence growth at rates of −1.26, 195.73, and −94.48%, respectively. For GBD regions, the most pronounced contributions to incidence growth from aging, population growth, and epidemiological changes occurred in Central Latin America (338.4%), Central Asia (1,444.51%), and Central Latin America (1,349.74%), respectively. For the global increase in YLDs, aging, population growth, and epidemiological changes account for 40.14, 111.27, and −51.41%, respectively. Within GBD regions, the most notable contributions to YLDs growth from aging, population growth, and epidemiological changes were observed in High-income Asia Pacific (552.73%), Central Asia (286.56%), and Central Europe (141.9%), respectively.

## 4 Discussion

The current study presents the most comprehensive global assessment of the forearm fractures from 1990 to 2021, offering valuable insights into the evolving disease burden. Forearm fractures are among the most common types of fractures, posing a significant threat to people’s health and wellbeing and imposing a substantial burden on healthcare systems. Therefore, investigating the temporal trends, geographic distribution, and demographic factors of forearm fractures is of great importance for policymakers and health-related sectors to develop targeted measures to address this issue. The current study found that although incidence and YLDs rates have declined or stabilized, absolute numbers continue to rise. The incidence rate of forearm fractures exhibits a bimodal distribution, peaking in youth and older adulthood. The higher burden among females, particularly those who are postmenopausal, aligns with known associations between osteoporosis and fracture risk. In recent years, the burden has shown a trend toward aging populations. Falls, road injuries, and exposure to mechanical forces were the three leading causes of forearm fractures, with falls being the most prominent.

This study also revealed variations in the geographic distribution of the disease burden of forearm fractures, which may be associated with climatic factors. In cold regions like Russia, icy and slippery ground conditions can increase the risk of forearm fracture caused by falling, especially for the older adult. On the other hand, cold temperatures can also negatively impact essential physical functions necessary for independent living among older individuals (16). Literature has identified winter as the peak season for forearm fractures in the older adult (10, 17), corroborating this idea. Besides, a Norwegian epidemiologic osteoporosis study showed that under conditions of low temperature, the risk of forearm fracture was augmented by as much as 53% (18).

We identified a bimodal age distribution, with high incidence rates among youth and older adults. Previous studies have shown that forearm fractures are a common type of fracture among adolescents (19). High levels of physical activity and participation in sports or manual labor during youth and adolescence, particularly among males, contribute to the risk of forearm fractures. Implementing safety education, promoting the use of protective equipment, and ensuring safe environments can help reduce the occurrence of forearm fractures in younger individuals.

The older adult population is another high-risk group. Our study found that due to population aging and structural changes in the disease pattern of forearm fractures, the older adult, especially women, will require particular attention. Previous literature has demonstrated that the incidence of forearm fractures in women significantly increases after menopause (6, 7, 10). Women tend to have a more delicate bone structure and lower bone density compared to men, making them more susceptible to forearm fractures when subjected to external forces. Additionally, postmenopausal women experience a significant decline in bone density, resulting in more severe osteoporosis and an increased risk of forearm fractures. Even minor falls can lead to forearm fractures in this population. Therefore, it is essential to implement fall prevention interventions, osteoporosis screening, and educational programs focusing on lifestyle factors for the older adult. There is a need to enhance bone mineral density assessment and management for postmenopausal women (20). Additionally, research has found that older adults who experience forearm fracture are at high risk of subsequent fractures (21); however, the prescription rate for anti-osteoporosis medications after forearm fractures in older adult patients is low, possibly due to overlooked assessments (22, 23). One previous study in France indicated that only 10% of women aged 50 and older underwent bone density assessment after a forearm fracture (24). Overall, in addition to orthopedic treatments, comprehensive osteoporosis assessments and continued follow-up are crucial for older adult patients with forearm fractures.

In our current study, we confirmed that the leading cause of forearm fractures is falls. Previous literature has also reported a strong association between falls and forearm fractures (25–27). Falls result in significant health losses and pose a substantial global burden (28). Previous studies have shown that in 2014, 28.7% of community-dwelling individuals aged 65 and older have experienced at least one fall per year, with an average of 0.67 falls per person-year (29). Falls among the older adult are a significant public health concern, resulting in considerable socioeconomic costs (30). With the increasing aging population, healthcare system faces a significant challenge regarding falls in older adults. The risk of falling is high among the older adult (31), which is associated with weakness and declining physical functions. Even minor falls can lead to osteoporotic fracture. Multifactorial fall risk assessments and targeted multi-component interventions are necessary to prevent falls in this population (32–34). These require interventions from physiological, psychological, and social aspects, as well as the active involvement and collaboration of policymakers, healthcare workers, and community managers, to establish a comprehensive fall prevention system for the older adult.

## 5 Limitations

While the GBD 2021 represents a robust platform, it is not without limitations. Firstly, the GBD 2021 relies on multiple data sources that vary by country. Although this provides broad coverage, data quality and availability may differ by region; specifically, low-income countries may have sparse or lacking data, potentially affecting the accuracy. Secondly, the incidence and YLDs values for forearm fractures typically carry a degree of uncertainty. The GBD 2021 uses sophisticated modeling techniques to address these uncertainties, but some residual errors remain and need to be considered when interpreting our results. Thirdly, the GBD 2021 provides burden estimates disaggregated by age, gender, and geographic location; however, other demographic factors, such as race/ethnicity, may also influence the risk and outcomes of fractures. The potential impact of these factors may not be fully addressed, and they could be important in interpreting the results. Fourth, the impact of the COVID-19 pandemic was not addressed in the current study; however, we did identify an increasing burden in forearm fractures during the COVID-19 pandemic, which may have been related to restrictions on hospitalization and outpatient visits.

## 6 Conclusion

The incidence and YLDs number of forearm fractures have increased since 1990, with a higher burden among females. The incidence rate of forearm fractures displays a bimodal distribution, peaking in youth and older adults. The age pattern exhibits a trend toward aging populations. Falls continue to demonstrate the leading cause of forearm fractures, and the disease burden they impose is the most prominent among older adult females.

## Data Availability

Publicly available datasets were analyzed in this study. This data can be found here at: https://vizhub.healthdata.org/gbd-results/.

## Footnotes

^1^ https://vizhub.healthdata.org/gbd-results/
