# Supplementary material for: Global, regional, and national burden of forearm fracture from 1990 to 2021: a study based on GBD 2021
Source: Front Public Health. 2025 Jul 25;13:1638277. doi: 10.3389/fpubh.2025.1638277 (PMC12332770; doi:10.3389/fpubh.2025.1638277)
Supplement: Supplementary file 1 [file Table_1.docx]

**Global, regional, and national burden of forearm fracture from 1990 to 2021: a study based on GBD 2021**

**Supplementary Material**

**Contents**

**Table S1………………………………………………………………………………………. 2**

**Table S2……………………………………………………………………………….……… 5**

**Table S3………………………………………………………………………………………. 8**

**Table S4………………………………………………………………………………………. 32**

Table S1. Incidence of forearm fracture in 1990 and 2021 for both sexes in globe and 21 GBD regions, with EAPC from 1990 and 2021.

| **Location** | **Number in 1990 (95% UI)** | **Number in 2021 (95% UI)** | **Number change rate (95% UI)** | **CR in 1990 (95% UI)** | **CR in 2021 (95% UI)** | **EAPC of CR, % per year (95% CI)** | **ASR in 1990 (95% UI)** | **ASR in 2021 (95% UI)** | **EAPC of ASR, % per year (95% CI)** |
| --- | --- | --- | --- | --- | --- | --- | --- | --- | --- |
| Global | 26098810 (20967988 to 32372267) | 31905396 (25403829 to 39982115) | 0.22 (0.16 to 0.27) | 489.33 (393.13 to 606.95) | 404.31 (321.92 to 506.66) | -0.74 (-0.79 to -0.69) | 483.28 (387.42 to 599.37) | 402.35 (319.86 to 505.21) | -0.7 (-0.76 to -0.65) |
| Andean Latin America | 174473 (145048 to 209115) | 268722 (216275 to 327400) | 0.54 (0.39 to 0.66) | 459.22 (381.77 to 550.4) | 406.33 (327.03 to 495.06) | -0.34 (-0.4 to -0.28) | 430.13 (359.45 to 511.77) | 399.69 (321.22 to 485.96) | -0.18 (-0.24 to -0.13) |
| Australasia | 121939 (89712 to 157537) | 174378 (128593 to 231022) | 0.43 (0.34 to 0.51) | 601.39 (442.45 to 776.95) | 563.21 (415.33 to 746.16) | -0.15 (-0.25 to -0.05) | 624.78 (457.74 to 815.98) | 594.58 (428.55 to 792.88) | -0.12 (-0.27 to 0.03) |
| Caribbean | 138314 (115038 to 164870) | 205406 (169932 to 246153) | 0.49 (0.4 to 0.62) | 391.91 (325.96 to 467.15) | 432.81 (358.06 to 518.67) | 0.28 (-0.42 to 0.97) | 382.94 (319.93 to 456.95) | 436.1 (362.5 to 523.7) | 0.35 (-0.35 to 1.06) |
| Central Asia | 485675 (395740 to 588023) | 496808 (403576 to 601441) | 0.02 (0 to 0.05) | 700.71 (570.96 to 848.37) | 518.54 (421.23 to 627.75) | -1.08 (-1.17 to -1) | 655.28 (536.18 to 791.55) | 515.48 (417.89 to 624.25) | -0.87 (-0.96 to -0.79) |
| Central Europe | 1453791 (1170269 to 1763969) | 986092 (785348 to 1214142) | -0.32 (-0.35 to -0.3) | 1162.18 (935.53 to 1410.14) | 855.51 (681.35 to 1053.36) | -1.14 (-1.22 to -1.06) | 1178.55 (947.86 to 1430.49) | 909.72 (720.21 to 1119.3) | -0.94 (-1.01 to -0.87) |
| Central Latin America | 1285069 (1016928 to 1602942) | 1249753 (995244 to 1539091) | -0.03 (-0.06 to 0.01) | 781.63 (618.54 to 974.98) | 493.97 (393.37 to 608.33) | -0.86 (-1.15 to -0.57) | 719.97 (575.67 to 884.86) | 499.4 (396.56 to 617.5) | -0.56 (-0.85 to -0.27) |
| Central Sub-Saharan Africa | 144757 (120548 to 175757) | 308510 (256018 to 376675) | 1.13 (1.02 to 1.24) | 263.37 (219.33 to 319.77) | 225.31 (186.97 to 275.09) | -0.99 (-1.39 to -0.6) | 248.7 (208.91 to 298.57) | 225.4 (187.62 to 270.76) | -0.75 (-1.1 to -0.39) |
| East Asia | 4527959 (3647766 to 5653521) | 5901599 (4594522 to 7549669) | 0.3 (0.21 to 0.4) | 371.92 (299.63 to 464.38) | 400.71 (311.96 to 512.62) | -0.16 (-0.54 to 0.23) | 368.48 (296.32 to 459.54) | 397.63 (312.39 to 507.23) | -0.23 (-0.63 to 0.16) |
| Eastern Europe | 2597606 (2129496 to 3149138) | 1830109 (1476289 to 2262577) | -0.3 (-0.33 to -0.26) | 1146.89 (940.21 to 1390.4) | 885.14 (714.01 to 1094.31) | -1.15 (-1.49 to -0.81) | 1176.39 (955.05 to 1436.72) | 943.29 (755.11 to 1173.17) | -1.02 (-1.29 to -0.75) |
| Eastern Sub-Saharan Africa | 655378 (512060 to 922409) | 938523 (777063 to 1144677) | 0.43 (0.12 to 0.69) | 343.44 (268.34 to 483.38) | 220.26 (182.37 to 268.64) | -1.21 (-1.46 to -0.97) | 319.56 (253.53 to 429.93) | 221.73 (185.08 to 265.61) | -1 (-1.22 to -0.79) |
| High-income Asia Pacific | 730922 (546225 to 957520) | 559474 (411483 to 742966) | -0.23 (-0.28 to -0.18) | 421.57 (315.04 to 552.27) | 301.69 (221.89 to 400.64) | -1.26 (-1.37 to -1.16) | 427.86 (319.88 to 560.67) | 313.44 (228.19 to 416.96) | -1.16 (-1.29 to -1.04) |
| High-income North America | 1031139 (773113 to 1344019) | 1336445 (998791 to 1792531) | 0.3 (0.18 to 0.43) | 366.42 (274.73 to 477.6) | 361.03 (269.82 to 484.24) | -0.09 (-0.29 to 0.11) | 362.07 (271.48 to 471.84) | 306.6 (228.67 to 402.31) | -0.66 (-0.87 to -0.45) |
| North Africa and Middle East | 2000794 (1662872 to 2376440) | 3093154 (2534054 to 3770699) | 0.55 (0.44 to 0.66) | 589.87 (490.25 to 700.62) | 496.49 (406.75 to 605.25) | -0.33 (-0.41 to -0.26) | 546.4 (458.09 to 646.85) | 486.29 (399.4 to 591.02) | -0.15 (-0.22 to -0.08) |
| Oceania | 19396 (15756 to 24078) | 48970 (39493 to 61705) | 1.52 (1.37 to 1.66) | 296.12 (240.56 to 367.61) | 351.6 (283.56 to 443.04) | 0.17 (-0.32 to 0.66) | 296.47 (242.07 to 364.94) | 357.76 (287.63 to 449.57) | 0.27 (-0.15 to 0.7) |
| South Asia | 4918976 (3937728 to 6230424) | 7486598 (5984746 to 9493063) | 0.52 (0.43 to 0.61) | 449.87 (360.13 to 569.81) | 405.43 (324.1 to 514.09) | -0.55 (-0.66 to -0.43) | 463.4 (371.28 to 583.13) | 412.64 (327.21 to 524.1) | -0.56 (-0.66 to -0.46) |
| Southeast Asia | 1758877 (1459674 to 2089414) | 2158505 (1756110 to 2637723) | 0.23 (0.15 to 0.3) | 377.84 (313.57 to 448.85) | 309.11 (251.48 to 377.73) | -0.67 (-0.89 to -0.45) | 370.06 (308.55 to 439.02) | 310.83 (253.13 to 380.06) | -0.58 (-0.8 to -0.35) |
| Southern Latin America | 177637 (134263 to 227368) | 240010 (179411 to 308000) | 0.35 (0.3 to 0.4) | 358.58 (271.03 to 458.97) | 354.55 (265.03 to 454.99) | -0.03 (-0.22 to 0.15) | 354.82 (267.79 to 453.92) | 366.66 (272.14 to 471.41) | 0.14 (-0.04 to 0.33) |
| Southern Sub-Saharan Africa | 182278 (153615 to 215465) | 204372 (172550 to 244390) | 0.12 (0.09 to 0.16) | 347.74 (293.06 to 411.05) | 254.5 (214.87 to 304.33) | -1.12 (-1.26 to -0.99) | 340.93 (286.45 to 405.76) | 247.45 (209.04 to 294.67) | -1.17 (-1.3 to -1.05) |
| Tropical Latin America | 1246155 (985576 to 1578313) | 1323439 (1052472 to 1654671) | 0.06 (0.01 to 0.12) | 816.87 (646.06 to 1034.61) | 581.67 (462.57 to 727.25) | -1.24 (-1.45 to -1.02) | 769.6 (612.23 to 972.04) | 585.42 (462.38 to 738.52) | -0.98 (-1.19 to -0.77) |
| Western Europe | 1965226 (1415075 to 2630037) | 1989413 (1401961 to 2695634) | 0.01 (-0.04 to 0.06) | 511.23 (368.11 to 684.17) | 454.84 (320.53 to 616.3) | -0.38 (-0.44 to -0.32) | 515.7 (371.76 to 691.39) | 458.91 (323.65 to 631.21) | -0.37 (-0.48 to -0.26) |
| Western Sub-Saharan Africa | 482448 (396750 to 582283) | 1105118 (907977 to 1353067) | 1.29 (1.2 to 1.38) | 249.77 (205.4 to 301.45) | 225.61 (185.37 to 276.23) | -0.38 (-0.45 to -0.32) | 238.57 (199.83 to 285.62) | 225.5 (186.58 to 272.76) | -0.23 (-0.29 to -0.17) |

CR, crude rate; ASR, age-standardized rate; EAPC, estimated annual percentage change; UI, uncertainty interval; CI, confidence interval.

Table S2. YLDs of forearm fracture in 1990 and 2021 for both sexes in globe and 21 GBD regions, with EAPC from 1990 and 2021.

| **Location** | **Number in 1990 (95% UI)** | **Number in 2021 (95% UI)** | **Number change rate (95% UI)** | **CR in 1990 (95% UI)** | **CR in 2021 (95% UI)** | **EAPC of CR, % per year (95% CI)** | **ASR in 1990 (95% UI)** | **ASR in 2021 (95% UI)** | **EAPC of ASR, % per year (95% CI)** |
| --- | --- | --- | --- | --- | --- | --- | --- | --- | --- |
| Global | 144166 (87129 to 229017) | 205031 (126061 to 320235) | 0.42 (0.37 to 0.48) | 2.7 (1.63 to 4.29) | 2.6 (1.6 to 4.06) | -0.26 (-0.32 to -0.21) | 2.98 (1.82 to 4.7) | 2.51 (1.54 to 3.93) | -0.68 (-0.73 to -0.63) |
| Andean Latin America | 817 (485 to 1341) | 1463 (877 to 2324) | 0.79 (0.65 to 0.93) | 2.15 (1.28 to 3.53) | 2.21 (1.33 to 3.51) | 0.16 (0.1 to 0.22) | 2.37 (1.44 to 3.83) | 2.24 (1.35 to 3.55) | -0.17 (-0.23 to -0.1) |
| Australasia | 732 (444 to 1163) | 1222 (743 to 1898) | 0.67 (0.56 to 0.81) | 3.61 (2.19 to 5.73) | 3.95 (2.4 to 6.13) | 0.38 (0.27 to 0.49) | 3.53 (2.14 to 5.63) | 3.35 (1.98 to 5.37) | -0.1 (-0.23 to 0.03) |
| Caribbean | 739 (444 to 1208) | 1286 (807 to 2008) | 0.74 (0.63 to 0.88) | 2.09 (1.26 to 3.42) | 2.71 (1.7 to 4.23) | 0.87 (0.42 to 1.32) | 2.28 (1.38 to 3.68) | 2.58 (1.61 to 4.06) | 0.4 (-0.05 to 0.86) |
| Central Asia | 2362 (1408 to 3800) | 2648 (1603 to 4176) | 0.12 (0.08 to 0.18) | 3.41 (2.03 to 5.48) | 2.76 (1.67 to 4.36) | -0.74 (-0.81 to -0.67) | 3.59 (2.18 to 5.69) | 2.82 (1.71 to 4.44) | -0.83 (-0.89 to -0.77) |
| Central Europe | 8611 (5232 to 13746) | 7008 (4328 to 10975) | -0.19 (-0.22 to -0.15) | 6.88 (4.18 to 10.99) | 6.08 (3.76 to 9.52) | -0.51 (-0.57 to -0.45) | 6.62 (4.01 to 10.67) | 4.99 (2.99 to 8.05) | -1.02 (-1.09 to -0.96) |
| Central Latin America | 6120 (3597 to 10050) | 7258 (4378 to 11524) | 0.19 (0.13 to 0.27) | 3.72 (2.19 to 6.11) | 2.87 (1.73 to 4.55) | -0.27 (-0.53 to -0.02) | 4.15 (2.52 to 6.68) | 2.87 (1.73 to 4.56) | -0.66 (-0.9 to -0.41) |
| Central Sub-Saharan Africa | 692 (409 to 1164) | 1536 (916 to 2491) | 1.22 (1.09 to 1.38) | 1.26 (0.74 to 2.12) | 1.12 (0.67 to 1.82) | -0.74 (-1.02 to -0.46) | 1.56 (0.95 to 2.49) | 1.48 (0.91 to 2.33) | -0.42 (-0.61 to -0.23) |
| East Asia | 26379 (15830 to 41353) | 43389 (27324 to 67042) | 0.64 (0.54 to 0.76) | 2.17 (1.3 to 3.4) | 2.95 (1.86 to 4.55) | 0.63 (0.27 to 1) | 2.42 (1.48 to 3.78) | 2.51 (1.53 to 3.93) | -0.3 (-0.65 to 0.05) |
| Eastern Europe | 15724 (9458 to 25074) | 12589 (7674 to 19662) | -0.2 (-0.23 to -0.16) | 6.94 (4.18 to 11.07) | 6.09 (3.71 to 9.51) | -0.76 (-1.09 to -0.42) | 6.58 (3.95 to 10.58) | 5.25 (3.12 to 8.3) | -1.05 (-1.34 to -0.76) |
| Eastern Sub-Saharan Africa | 2852 (1629 to 4883) | 4534 (2690 to 7343) | 0.59 (0.3 to 0.79) | 1.49 (0.85 to 2.56) | 1.06 (0.63 to 1.72) | -0.99 (-1.17 to -0.81) | 1.78 (1.06 to 2.87) | 1.4 (0.87 to 2.18) | -0.72 (-0.84 to -0.59) |
| High-income Asia Pacific | 4614 (2807 to 7390) | 4836 (3074 to 7479) | 0.05 (-0.02 to 0.13) | 2.66 (1.62 to 4.26) | 2.61 (1.66 to 4.03) | -0.24 (-0.35 to -0.12) | 2.53 (1.53 to 4.06) | 1.86 (1.13 to 3) | -1.16 (-1.29 to -1.03) |
| High-income North America | 7116 (4405 to 11234) | 11101 (6943 to 17210) | 0.56 (0.46 to 0.69) | 2.53 (1.57 to 3.99) | 3 (1.88 to 4.65) | 0.52 (0.33 to 0.71) | 2.3 (1.42 to 3.68) | 2.1 (1.29 to 3.33) | -0.37 (-0.54 to -0.2) |
| North Africa and Middle East | 9391 (5562 to 15249) | 16298 (9855 to 26127) | 0.74 (0.64 to 0.83) | 2.77 (1.64 to 4.5) | 2.62 (1.58 to 4.19) | -0.05 (-0.1 to -0.01) | 3.08 (1.86 to 4.9) | 2.75 (1.67 to 4.37) | -0.26 (-0.3 to -0.22) |
| Oceania | 97 (58 to 155) | 260 (155 to 406) | 1.67 (1.5 to 1.89) | 1.48 (0.89 to 2.37) | 1.87 (1.11 to 2.91) | 0.47 (0.12 to 0.81) | 1.91 (1.19 to 2.93) | 2.31 (1.41 to 3.56) | 0.4 (0.16 to 0.65) |
| South Asia | 24624 (14474 to 39226) | 44122 (26815 to 67448) | 0.79 (0.7 to 0.9) | 2.25 (1.32 to 3.59) | 2.39 (1.45 to 3.65) | 0.02 (-0.06 to 0.1) | 2.88 (1.75 to 4.55) | 2.7 (1.67 to 4.1) | -0.36 (-0.42 to -0.29) |
| Southeast Asia | 8871 (5242 to 14291) | 12986 (8010 to 20416) | 0.46 (0.4 to 0.54) | 1.91 (1.13 to 3.07) | 1.86 (1.15 to 2.92) | -0.15 (-0.28 to -0.02) | 2.25 (1.36 to 3.54) | 1.92 (1.19 to 3.02) | -0.57 (-0.7 to -0.45) |
| Southern Latin America | 996 (601 to 1585) | 1511 (914 to 2394) | 0.52 (0.42 to 0.63) | 2.01 (1.21 to 3.2) | 2.23 (1.35 to 3.54) | 0.34 (0.19 to 0.5) | 2.05 (1.25 to 3.26) | 2.09 (1.25 to 3.35) | 0.09 (-0.07 to 0.25) |
| Southern Sub-Saharan Africa | 1001 (605 to 1605) | 1210 (728 to 1914) | 0.21 (0.16 to 0.27) | 1.91 (1.15 to 3.06) | 1.51 (0.91 to 2.38) | -0.93 (-1.07 to -0.79) | 2.31 (1.43 to 3.59) | 1.61 (0.98 to 2.54) | -1.33 (-1.48 to -1.19) |
| Tropical Latin America | 6138 (3626 to 9642) | 8088 (4862 to 12531) | 0.32 (0.24 to 0.42) | 4.02 (2.38 to 6.32) | 3.55 (2.14 to 5.51) | -0.53 (-0.69 to -0.37) | 4.4 (2.65 to 6.84) | 3.39 (2.03 to 5.25) | -0.93 (-1.1 to -0.76) |
| Western Europe | 14002 (8593 to 21744) | 16412 (10265 to 25127) | 0.17 (0.13 to 0.23) | 3.64 (2.24 to 5.66) | 3.75 (2.35 to 5.74) | 0.1 (0.04 to 0.15) | 3.15 (1.9 to 5.04) | 2.76 (1.65 to 4.41) | -0.42 (-0.52 to -0.33) |
| Western Sub-Saharan Africa | 2288 (1354 to 3733) | 5276 (3081 to 8525) | 1.31 (1.24 to 1.37) | 1.18 (0.7 to 1.93) | 1.08 (0.63 to 1.74) | -0.37 (-0.43 to -0.32) | 1.46 (0.89 to 2.3) | 1.42 (0.86 to 2.21) | -0.16 (-0.21 to -0.11) |

YLDs, years lived with disability; CR, crude rate; ASR, age-standardized rate; EAPC, estimated annual percentage change; UI, uncertainty interval; CI, confidence interval.

Table S3. Incidence of forearm fracture in 1990 and 2021 for both sexes in countries and territories, with EAPC from 1990 to 2021.

| **Location** | **Number in 1990 (95% UI)** | **Number in 2021 (95% UI)** | **Number change rate (95% UI)** | **CR in 1990 (95% UI)** | **CR in 2021 (95% UI)** | **EAPC of CR, % per year (95% CI)** | **ASR in 1990 (95% UI)** | **ASR in 2021 (95% UI)** | **EAPC of ASR, % per year (95% CI)** |
| --- | --- | --- | --- | --- | --- | --- | --- | --- | --- |
| Afghanistan | 50208 (41703 to 60342) | 230302 (162790 to 383454) | 3.59 (2.46 to 5.7) | 504.94 (419.4 to 606.85) | 737.62 (521.39 to 1228.15) | 0.61 (0.12 to 1.11) | 477.52 (402.21 to 571.6) | 655.51 (473.59 to 1058.19) | 0.5 (0.05 to 0.94) |
| Albania | 35976 (28416 to 45094) | 22058 (17688 to 27292) | -0.39 (-0.43 to -0.34) | 1088.48 (859.75 to 1364.34) | 826.82 (663.01 to 1022.98) | -1.23 (-1.46 to -1.01) | 1019.08 (802.39 to 1278) | 919.21 (724.31 to 1145.64) | -0.66 (-0.86 to -0.46) |
| Algeria | 151450 (124650 to 184915) | 187245 (151873 to 231566) | 0.24 (0.16 to 0.31) | 598.9 (492.92 to 731.24) | 423.65 (343.62 to 523.93) | -1.33 (-1.44 to -1.23) | 549.71 (455.02 to 662.12) | 423.3 (342.69 to 525.47) | -1.04 (-1.12 to -0.96) |
| American Samoa | 151 (123 to 188) | 145 (116 to 180) | -0.04 (-0.09 to 0.01) | 311.45 (253.02 to 388.14) | 290.46 (233.44 to 361.84) | -0.14 (-0.87 to 0.59) | 305.75 (249.36 to 376.37) | 295.78 (238.79 to 370.02) | 0 (-0.7 to 0.71) |
| Andorra | 331 (233 to 450) | 590 (417 to 811) | 0.78 (0.64 to 0.91) | 609.4 (429.03 to 828.49) | 689.22 (487.19 to 947.08) | 0.38 (0.32 to 0.44) | 622.28 (442.45 to 846.2) | 654.31 (457.97 to 901.67) | 0.15 (0.07 to 0.24) |
| Angola | 35252 (27984 to 47857) | 71789 (59423 to 88919) | 1.04 (0.51 to 1.46) | 343.05 (272.31 to 465.7) | 219.5 (181.68 to 271.87) | -1.88 (-2.46 to -1.29) | 315.73 (254.92 to 417.04) | 220.39 (182.81 to 267.03) | -1.54 (-2.07 to -1) |
| Antigua and Barbuda | 229 (184 to 286) | 339 (264 to 428) | 0.48 (0.4 to 0.57) | 380.82 (305.55 to 474.93) | 378.73 (295.74 to 479.04) | -0.23 (-0.58 to 0.14) | 367.67 (296.27 to 457.89) | 395.46 (311.76 to 504.88) | 0.04 (-0.33 to 0.4) |
| Argentina | 113613 (85912 to 144259) | 146038 (110347 to 185795) | 0.29 (0.24 to 0.33) | 343.13 (259.47 to 435.68) | 321.05 (242.59 to 408.45) | -0.2 (-0.41 to 0.02) | 341.16 (258.37 to 434.08) | 329.38 (247.28 to 421.34) | -0.07 (-0.28 to 0.14) |
| Armenia | 27649 (22712 to 33612) | 11793 (9709 to 14094) | -0.57 (-0.6 to -0.55) | 808.31 (663.98 to 982.62) | 393.71 (324.16 to 470.54) | -2.19 (-2.64 to -1.73) | 782.6 (641.01 to 955.04) | 424.34 (345.52 to 510.57) | -1.78 (-2.29 to -1.27) |
| Australia | 98053 (72484 to 127426) | 142843 (104811 to 189244) | 0.46 (0.36 to 0.55) | 581.6 (429.94 to 755.83) | 553.83 (406.37 to 733.73) | -0.08 (-0.17 to 0.01) | 608.04 (446.23 to 796.46) | 579.67 (417.38 to 776.37) | -0.09 (-0.22 to 0.04) |
| Austria | 49543 (35848 to 65609) | 44631 (31787 to 60730) | -0.1 (-0.14 to -0.06) | 637.77 (461.47 to 844.59) | 496.88 (353.88 to 676.1) | -0.67 (-0.75 to -0.6) | 634.68 (458.37 to 843.97) | 505.85 (357.7 to 695.08) | -0.63 (-0.72 to -0.53) |
| Azerbaijan | 41130 (33933 to 49019) | 44157 (35736 to 53568) | 0.07 (0.03 to 0.12) | 561.39 (463.15 to 669.07) | 420.54 (340.34 to 510.16) | -1.06 (-1.31 to -0.81) | 520.69 (429.83 to 619.68) | 431.37 (348.54 to 524.76) | -0.71 (-0.99 to -0.42) |
| Bahrain | 2073 (1696 to 2496) | 5760 (4670 to 7081) | 1.78 (1.62 to 1.94) | 409.4 (334.91 to 492.73) | 376.54 (305.29 to 462.91) | -0.5 (-0.68 to -0.31) | 374.73 (307.36 to 448.75) | 368.1 (298.81 to 456.75) | -0.31 (-0.46 to -0.16) |
| Bangladesh | 269501 (218549 to 335060) | 402372 (317710 to 512391) | 0.49 (0.37 to 0.62) | 246.97 (200.28 to 307.05) | 244.4 (192.98 to 311.23) | -0.79 (-1.75 to 0.18) | 223.72 (182.75 to 276.78) | 236.01 (187.22 to 299.71) | -0.5 (-1.39 to 0.39) |
| Barbados | 719 (596 to 873) | 821 (675 to 1003) | 0.14 (0.09 to 0.2) | 283.55 (235.27 to 344.44) | 274.56 (225.63 to 335.38) | -0.22 (-0.36 to -0.08) | 283.02 (234.16 to 345.9) | 299.54 (243.99 to 368.59) | 0.07 (-0.09 to 0.23) |
| Belarus | 105234 (85450 to 129030) | 89058 (70288 to 111900) | -0.15 (-0.2 to -0.11) | 1007.56 (818.14 to 1235.4) | 955.08 (753.79 to 1200.04) | -0.25 (-0.6 to 0.11) | 1037.08 (839.27 to 1274.94) | 1015.61 (803.28 to 1282.85) | -0.16 (-0.45 to 0.13) |
| Belgium | 57833 (41477 to 78350) | 71876 (50709 to 97505) | 0.24 (0.16 to 0.31) | 579.56 (415.65 to 785.16) | 626.68 (442.13 to 850.14) | 0.45 (0.15 to 0.75) | 593.12 (428.49 to 803.34) | 589.23 (413.42 to 802.89) | 0.2 (-0.22 to 0.61) |
| Belize | 692 (566 to 837) | 1693 (1400 to 2054) | 1.45 (1.28 to 1.61) | 370.06 (302.65 to 447.84) | 394.66 (326.21 to 478.65) | 0.12 (-0.04 to 0.29) | 332.18 (275.71 to 398.76) | 376.91 (311.47 to 456.58) | 0.27 (0.1 to 0.45) |
| Benin | 12253 (10175 to 14706) | 31353 (25854 to 38144) | 1.56 (1.43 to 1.67) | 252.61 (209.77 to 303.17) | 232.26 (191.52 to 282.56) | -0.31 (-0.36 to -0.25) | 236.59 (198.41 to 281.03) | 229.73 (191.65 to 275.24) | -0.15 (-0.2 to -0.09) |
| Bermuda | 181 (150 to 219) | 186 (150 to 227) | 0.02 (-0.04 to 0.1) | 305.45 (252.86 to 368.24) | 292.13 (236.55 to 357.54) | -0.18 (-0.3 to -0.06) | 316.36 (259.55 to 384.75) | 329.22 (263.94 to 402.01) | 0.09 (-0.04 to 0.22) |
| Bhutan | 1884 (1492 to 2309) | 2797 (2237 to 3501) | 0.48 (0.36 to 0.62) | 299.02 (236.83 to 366.48) | 369.52 (295.53 to 462.57) | 0.42 (0.01 to 0.83) | 291.04 (235.59 to 351.08) | 376.69 (301.54 to 472.47) | 0.58 (0.2 to 0.96) |
| Bosnia and Herzegovina | 45568 (35972 to 57149) | 23313 (18503 to 28675) | -0.49 (-0.53 to -0.44) | 1013 (799.69 to 1270.46) | 705.83 (560.22 to 868.2) | -1.57 (-1.96 to -1.18) | 1001.82 (789.21 to 1252.41) | 817.58 (639.79 to 1012.75) | -1.12 (-1.52 to -0.72) |
| Botswana | 3320 (2741 to 3981) | 6475 (5450 to 7734) | 0.95 (0.84 to 1.07) | 251.65 (207.8 to 301.77) | 270.55 (227.74 to 323.17) | 0.13 (-0.04 to 0.3) | 246.26 (205.9 to 292.23) | 263.7 (221.31 to 315.26) | 0.08 (-0.07 to 0.23) |
| Brazil | 1221434 (966028 to 1546937) | 1286168 (1022839 to 1608154) | 0.05 (0 to 0.11) | 822.47 (650.48 to 1041.65) | 583.68 (464.18 to 729.8) | -1.25 (-1.46 to -1.03) | 775 (616.86 to 978.6) | 587.89 (464.41 to 741.91) | -0.99 (-1.2 to -0.78) |
| Brunei Darussalam | 1089 (830 to 1406) | 1578 (1188 to 2050) | 0.45 (0.37 to 0.52) | 420.21 (320.2 to 542.14) | 349.78 (263.41 to 454.35) | -0.58 (-0.63 to -0.54) | 397.24 (302.6 to 512.25) | 345.29 (257.94 to 447.13) | -0.47 (-0.51 to -0.42) |
| Bulgaria | 99706 (80013 to 120508) | 57315 (45312 to 70166) | -0.43 (-0.46 to -0.39) | 1148.68 (921.81 to 1388.33) | 844.5 (667.63 to 1033.84) | -1.13 (-1.17 to -1.09) | 1246.83 (995.47 to 1512.01) | 1004.3 (788.34 to 1234.33) | -0.78 (-0.81 to -0.75) |
| Burkina Faso | 24010 (19888 to 28789) | 59661 (48889 to 72107) | 1.48 (1.31 to 1.74) | 251.99 (208.73 to 302.15) | 262.12 (214.79 to 316.8) | 0.06 (-0.04 to 0.16) | 242.73 (204.14 to 287.56) | 257.19 (213.85 to 308.74) | 0.14 (0.06 to 0.23) |
| Burundi | 13874 (11510 to 16634) | 28147 (23083 to 34379) | 1.03 (0.94 to 1.12) | 249.83 (207.25 to 299.54) | 212.91 (174.6 to 260.04) | -3.29 (-4.87 to -1.67) | 242.51 (201.28 to 288.23) | 209.97 (173.11 to 252.83) | -2.99 (-4.41 to -1.55) |
| Cambodia | 38155 (31340 to 46303) | 64856 (51576 to 82506) | 0.7 (0.52 to 0.86) | 371.42 (305.08 to 450.73) | 380.52 (302.6 to 484.07) | 0.04 (-0.11 to 0.19) | 361.64 (298.18 to 434.41) | 386.58 (305.78 to 491.63) | 0.19 (0.06 to 0.33) |
| Cameroon | 23142 (19066 to 28275) | 73213 (59730 to 90464) | 2.16 (1.97 to 2.37) | 221.73 (182.68 to 270.92) | 230.38 (187.95 to 284.66) | 0.18 (0.13 to 0.23) | 219.6 (181.15 to 265.7) | 231 (190.82 to 281.27) | 0.2 (0.16 to 0.24) |
| Canada | 89631 (67635 to 116442) | 133923 (98662 to 182034) | 0.49 (0.36 to 0.62) | 328.89 (248.18 to 427.27) | 357.42 (263.32 to 485.82) | 0.46 (0.39 to 0.53) | 317.4 (239.89 to 411.9) | 292.63 (217.55 to 387.94) | -0.14 (-0.2 to -0.09) |
| Central African Republic | 6754 (5700 to 7981) | 16557 (13421 to 21767) | 1.45 (1.1 to 2.2) | 247.34 (208.74 to 292.27) | 301.91 (244.72 to 396.9) | 0.73 (0.28 to 1.18) | 241.37 (203.35 to 284.4) | 286.18 (236.12 to 362.93) | 0.63 (0.24 to 1.01) |
| Chad | 15976 (13069 to 20022) | 43732 (36205 to 52903) | 1.74 (1.53 to 1.9) | 265.1 (216.85 to 332.24) | 246.39 (203.98 to 298.06) | -0.26 (-0.52 to 0) | 245.1 (202.86 to 300.87) | 236.48 (199.16 to 281.37) | -0.12 (-0.35 to 0.11) |
| Chile | 51044 (38329 to 65832) | 80916 (59167 to 106208) | 0.59 (0.5 to 0.68) | 384.2 (288.5 to 495.51) | 430.45 (314.76 to 565) | 0.37 (0.21 to 0.53) | 374.92 (281.28 to 483.3) | 455.57 (331.25 to 599) | 0.71 (0.52 to 0.91) |
| China | 4386645 (3532947 to 5478662) | 5790636 (4502029 to 7414711) | 0.32 (0.22 to 0.42) | 372.87 (300.3 to 465.69) | 407 (316.43 to 521.15) | -0.12 (-0.51 to 0.27) | 369.63 (296.7 to 460.96) | 404.52 (317.08 to 516.51) | -0.19 (-0.59 to 0.21) |
| Colombia | 227072 (185355 to 278808) | 208909 (167564 to 257185) | -0.08 (-0.13 to -0.03) | 698.87 (570.47 to 858.1) | 425.82 (341.55 to 524.22) | -1.73 (-1.79 to -1.67) | 649.84 (531.64 to 793.03) | 449.69 (355.92 to 563.28) | -1.28 (-1.33 to -1.23) |
| Comoros | 1080 (903 to 1305) | 1536 (1279 to 1848) | 0.42 (0.36 to 0.49) | 233.54 (195.16 to 282.03) | 206.41 (171.78 to 248.2) | -0.36 (-0.52 to -0.19) | 228.12 (192.2 to 268.76) | 209.25 (175.92 to 249.18) | -0.23 (-0.38 to -0.09) |
| Congo | 5596 (4670 to 6697) | 10792 (8987 to 13165) | 0.93 (0.84 to 1.02) | 233 (194.44 to 278.83) | 200.16 (166.69 to 244.18) | -1.4 (-2.4 to -0.4) | 234.15 (196.95 to 277.39) | 204.78 (171.29 to 248.98) | -1.22 (-2.09 to -0.34) |
| Cook Islands | 62 (50 to 77) | 53 (41 to 68) | -0.15 (-0.28 to -0.06) | 327.89 (262.65 to 407.8) | 296.82 (229.85 to 383.21) | -0.27 (-0.94 to 0.39) | 316.82 (255.26 to 389.73) | 307.34 (236.52 to 396.93) | -0.03 (-0.7 to 0.63) |
| Costa Rica | 16927 (12996 to 21882) | 21439 (16839 to 27308) | 0.27 (0.19 to 0.34) | 556.5 (427.26 to 719.4) | 451.55 (354.67 to 575.15) | -0.77 (-0.83 to -0.71) | 524.28 (405.85 to 669.53) | 477.45 (371.4 to 609.13) | -0.32 (-0.4 to -0.25) |
| Croatia | 56354 (45467 to 68304) | 42849 (33154 to 54699) | -0.24 (-0.33 to -0.16) | 1159.21 (935.26 to 1405.03) | 1018.12 (787.75 to 1299.69) | -0.52 (-0.66 to -0.38) | 1188.43 (956.66 to 1445.48) | 956.01 (734.01 to 1203.83) | -0.84 (-1 to -0.67) |
| Cuba | 49140 (40351 to 59058) | 61214 (48306 to 76065) | 0.25 (0.14 to 0.36) | 453.03 (372 to 544.46) | 543.18 (428.64 to 674.96) | 0.52 (0.48 to 0.57) | 447.43 (364.62 to 537.57) | 504.61 (406.18 to 621.2) | 0.32 (0.21 to 0.43) |
| Cyprus | 3892 (2827 to 5153) | 6270 (4455 to 8460) | 0.61 (0.5 to 0.71) | 500.17 (363.38 to 662.32) | 461.79 (328.08 to 623.08) | -0.25 (-0.45 to -0.05) | 507.63 (368.23 to 673.82) | 486.08 (344.6 to 662.26) | -0.08 (-0.28 to 0.11) |
| Democratic People's Republic of Korea | 53883 (44913 to 65134) | 55179 (46299 to 65895) | 0.02 (-0.03 to 0.09) | 261.68 (218.12 to 316.31) | 209.07 (175.43 to 249.67) | -0.59 (-0.68 to -0.5) | 257.41 (214.32 to 312.85) | 200.25 (168.75 to 237.74) | -0.7 (-0.78 to -0.62) |
| Democratic Republic of the Congo | 93632 (78314 to 111597) | 202383 (167516 to 245283) | 1.16 (1.02 to 1.31) | 245.45 (205.29 to 292.54) | 224.83 (186.09 to 272.48) | -0.75 (-1.23 to -0.27) | 231.85 (193.98 to 275.7) | 224.82 (187.32 to 268.97) | -0.52 (-0.94 to -0.09) |
| Denmark | 26750 (19314 to 35789) | 23751 (16625 to 32305) | -0.11 (-0.16 to -0.07) | 520.05 (375.49 to 695.79) | 405.88 (284.11 to 552.05) | -0.96 (-1.07 to -0.86) | 499.84 (354.14 to 668.55) | 425.22 (296.1 to 586.58) | -0.58 (-0.72 to -0.44) |
| Djibouti | 1097 (885 to 1386) | 2605 (2166 to 3116) | 1.37 (0.92 to 1.67) | 264.84 (213.54 to 334.69) | 206.94 (172.1 to 247.56) | -0.96 (-1.26 to -0.65) | 250.32 (207.12 to 308.01) | 214.11 (178.9 to 256.06) | -0.65 (-0.9 to -0.39) |
| Dominica | 213 (176 to 253) | 193 (161 to 230) | -0.09 (-0.14 to -0.05) | 294.34 (243.22 to 349.73) | 288.23 (240.1 to 342.93) | 0.25 (-0.18 to 0.69) | 280.6 (232.87 to 334.55) | 296.56 (245.51 to 351.87) | 0.55 (0.06 to 1.04) |
| Dominican Republic | 22563 (18775 to 26857) | 38851 (32237 to 46429) | 0.72 (0.65 to 0.8) | 315.53 (262.56 to 375.58) | 352.78 (292.72 to 421.59) | 0.37 (0.17 to 0.57) | 292.32 (243.5 to 347.84) | 348.25 (288.93 to 414.78) | 0.57 (0.37 to 0.77) |
| Ecuador | 46757 (38067 to 56887) | 84316 (68145 to 104205) | 0.8 (0.73 to 0.88) | 468.59 (381.5 to 570.12) | 466.73 (377.22 to 576.82) | -0.11 (-0.28 to 0.06) | 444.49 (364.99 to 535.03) | 457.27 (371.27 to 564.68) | 0.01 (-0.15 to 0.17) |
| Egypt | 280985 (234944 to 334423) | 396423 (327799 to 478410) | 0.41 (0.32 to 0.5) | 507.8 (424.6 to 604.38) | 375.3 (310.34 to 452.92) | -0.92 (-0.99 to -0.85) | 462.97 (387.94 to 550.72) | 366.3 (303.69 to 440.32) | -0.69 (-0.75 to -0.63) |
| El Salvador | 36673 (29299 to 47105) | 31579 (25695 to 39035) | -0.14 (-0.29 to -0.04) | 691.19 (552.21 to 887.82) | 489.57 (398.35 to 605.18) | -0.83 (-1 to -0.66) | 613.14 (494.18 to 770.58) | 484.66 (393.95 to 600.13) | -0.5 (-0.65 to -0.35) |
| Equatorial Guinea | 1029 (871 to 1225) | 2954 (2399 to 3634) | 1.87 (1.64 to 2.09) | 243.49 (205.89 to 289.84) | 195.3 (158.61 to 240.31) | -0.79 (-0.83 to -0.75) | 235.58 (198.29 to 278.3) | 200.25 (165.03 to 242.94) | -0.56 (-0.6 to -0.53) |
| Eritrea | 27676 (15504 to 57130) | 14481 (12102 to 17244) | -0.48 (-0.74 to -0.04) | 812.59 (455.22 to 1677.35) | 219.48 (183.42 to 261.36) | -2.01 (-3.13 to -0.88) | 701.18 (408.44 to 1370.58) | 228.94 (192.92 to 271.97) | -1.65 (-2.64 to -0.65) |
| Estonia | 19347 (15561 to 23912) | 9372 (7451 to 11744) | -0.52 (-0.54 to -0.5) | 1233.45 (992.08 to 1524.54) | 714.93 (568.39 to 895.92) | -2.09 (-2.22 to -1.96) | 1264.43 (1016.68 to 1562.28) | 787.5 (620.56 to 994.27) | -1.82 (-1.92 to -1.73) |
| Ethiopia | 280901 (196042 to 458910) | 257627 (206586 to 337991) | -0.08 (-0.37 to 0.2) | 555.48 (387.67 to 907.5) | 236.49 (189.64 to 310.26) | -1.94 (-2.49 to -1.4) | 513.98 (373.19 to 799.12) | 237.3 (193.9 to 299.02) | -1.79 (-2.25 to -1.33) |
| Fiji | 1917 (1555 to 2393) | 2082 (1693 to 2607) | 0.09 (0.03 to 0.14) | 252.78 (205.02 to 315.52) | 225.19 (183.17 to 282) | -0.53 (-0.7 to -0.35) | 240.15 (195.96 to 295.25) | 226.21 (184.84 to 282.11) | -0.32 (-0.48 to -0.16) |
| Finland | 34546 (24651 to 46378) | 34916 (24606 to 47305) | 0.01 (-0.04 to 0.06) | 689.55 (492.04 to 925.71) | 630.71 (444.48 to 854.5) | -0.45 (-0.93 to 0.03) | 707.36 (501.46 to 966.3) | 637.19 (444.61 to 886.28) | -0.45 (-0.98 to 0.08) |
| France | 357533 (260188 to 477916) | 379062 (268660 to 514773) | 0.06 (0 to 0.11) | 618.88 (450.38 to 827.26) | 570.96 (404.67 to 775.38) | -0.18 (-0.27 to -0.09) | 608.59 (441.75 to 809.04) | 543.59 (380.98 to 742.82) | -0.29 (-0.4 to -0.17) |
| Gabon | 2493 (2080 to 2996) | 4034 (3334 to 4925) | 0.62 (0.55 to 0.68) | 253.5 (211.5 to 304.66) | 222.14 (183.59 to 271.18) | -0.44 (-0.46 to -0.41) | 252.96 (211.24 to 301.69) | 227.36 (188.2 to 278.46) | -0.38 (-0.4 to -0.36) |
| Georgia | 43617 (34914 to 53477) | 27408 (22002 to 33742) | -0.37 (-0.4 to -0.34) | 789.72 (632.14 to 968.25) | 759.78 (609.93 to 935.39) | 0.01 (-0.19 to 0.22) | 808.59 (645.57 to 995.74) | 845.92 (677.03 to 1042.27) | 0.29 (0.07 to 0.51) |
| Germany | 386141 (278638 to 516560) | 387243 (274984 to 526756) | 0 (-0.05 to 0.05) | 483.03 (348.56 to 646.18) | 453.6 (322.1 to 617.01) | -0.25 (-0.31 to -0.18) | 493.8 (356.47 to 666.12) | 452.45 (318.3 to 618.88) | -0.32 (-0.4 to -0.25) |
| Ghana | 31954 (26267 to 38890) | 73207 (59530 to 90489) | 1.29 (1.19 to 1.4) | 213.43 (175.45 to 259.76) | 213.77 (173.83 to 264.23) | -0.03 (-0.11 to 0.04) | 211.8 (175.19 to 256.15) | 218.32 (178.56 to 266.76) | 0.04 (-0.03 to 0.11) |
| Greece | 53539 (38845 to 71480) | 35034 (25294 to 46408) | -0.35 (-0.37 to -0.32) | 515.33 (373.89 to 688.01) | 344.31 (248.59 to 456.1) | -1.33 (-1.44 to -1.22) | 538.34 (389.89 to 722.26) | 407.11 (290.87 to 553.32) | -0.88 (-1.01 to -0.74) |
| Greenland | 267 (201 to 349) | 254 (187 to 345) | -0.05 (-0.11 to 0.02) | 480.52 (361.34 to 629.05) | 452.97 (332.66 to 614.52) | -0.23 (-0.35 to -0.12) | 496.04 (372.76 to 652.7) | 418.13 (308.21 to 562.33) | -0.68 (-0.78 to -0.58) |
| Grenada | 306 (252 to 368) | 376 (302 to 462) | 0.23 (0.16 to 0.3) | 351.75 (289.25 to 422.65) | 366.72 (294.42 to 449.95) | 0.11 (-0.07 to 0.29) | 339.87 (281.12 to 407.69) | 371.75 (299.45 to 454.75) | 0.3 (0.12 to 0.47) |
| Guam | 393 (312 to 498) | 412 (327 to 516) | 0.05 (-0.01 to 0.11) | 287.02 (228.39 to 363.97) | 258.69 (205.3 to 324.39) | -0.36 (-0.49 to -0.22) | 275.85 (220.67 to 347.09) | 266.07 (210.22 to 336.73) | -0.14 (-0.28 to 0) |
| Guatemala | 57169 (45257 to 71330) | 93670 (75151 to 114947) | 0.64 (0.46 to 0.79) | 681.86 (539.78 to 850.76) | 594.08 (476.63 to 729.02) | -0.39 (-0.45 to -0.32) | 609.37 (492.41 to 754.64) | 572.28 (460.69 to 697.75) | -0.14 (-0.18 to -0.09) |
| Guinea | 15062 (12553 to 18188) | 31233 (25945 to 37821) | 1.07 (0.98 to 1.16) | 251.29 (209.42 to 303.44) | 232.57 (193.19 to 281.62) | -0.26 (-0.4 to -0.11) | 231.66 (194.64 to 274.04) | 227.75 (191.37 to 273.38) | -0.06 (-0.19 to 0.06) |
| Guinea-Bissau | 2829 (2373 to 3384) | 4772 (3985 to 5742) | 0.69 (0.62 to 0.76) | 280.92 (235.59 to 335.97) | 231.2 (193.07 to 278.23) | -0.86 (-1.18 to -0.54) | 271.35 (229.32 to 316.93) | 239.81 (202.96 to 287.28) | -0.59 (-0.85 to -0.32) |
| Guyana | 3018 (2520 to 3578) | 3299 (2755 to 3916) | 0.09 (0.04 to 0.15) | 387.05 (323.19 to 458.92) | 431.44 (360.29 to 512.02) | 0.17 (0.02 to 0.32) | 371.84 (310.37 to 439.38) | 426.63 (357.24 to 507.09) | 0.25 (0.1 to 0.4) |
| Haiti | 25762 (21589 to 30715) | 59441 (48536 to 76242) | 1.31 (0.99 to 1.95) | 403.74 (338.34 to 481.37) | 462.07 (377.3 to 592.67) | 0.09 (-1.41 to 1.6) | 377.79 (317.1 to 448) | 438.36 (359.66 to 557.43) | 0.18 (-1.32 to 1.71) |
| Honduras | 27507 (22123 to 34010) | 44824 (36846 to 54012) | 0.63 (0.54 to 0.71) | 584 (469.7 to 722.05) | 443.35 (364.45 to 534.24) | -1.27 (-1.89 to -0.64) | 499.79 (410.74 to 603.84) | 429.7 (352.37 to 516.18) | -0.84 (-1.44 to -0.24) |
| Hungary | 134633 (106297 to 166741) | 84938 (66335 to 105267) | -0.37 (-0.39 to -0.35) | 1295.18 (1022.58 to 1604.06) | 885.02 (691.19 to 1096.84) | -1.56 (-1.71 to -1.42) | 1247.75 (992.6 to 1534.28) | 904.53 (703.85 to 1129.62) | -1.27 (-1.4 to -1.15) |
| Iceland | 1266 (909 to 1703) | 1510 (1067 to 2055) | 0.19 (0.14 to 0.25) | 498.7 (357.96 to 670.78) | 430.95 (304.49 to 586.37) | -0.44 (-0.54 to -0.35) | 499.02 (356.64 to 674.41) | 456.59 (317.76 to 624.62) | -0.26 (-0.37 to -0.15) |
| India | 4270764 (3414498 to 5428599) | 6398991 (5082187 to 8144972) | 0.5 (0.4 to 0.59) | 500.64 (400.26 to 636.36) | 452.39 (359.29 to 575.82) | -0.48 (-0.55 to -0.42) | 520.29 (415.51 to 658.18) | 458.26 (361.37 to 586.83) | -0.55 (-0.61 to -0.49) |
| Indonesia | 692587 (564743 to 832366) | 750997 (615269 to 910997) | 0.08 (0.03 to 0.14) | 374.41 (305.29 to 449.97) | 269.26 (220.59 to 326.62) | -1.15 (-1.31 to -0.99) | 368.39 (301.04 to 443.99) | 276.11 (225.98 to 336.41) | -1.02 (-1.17 to -0.86) |
| Iraq | 137356 (112509 to 163921) | 220767 (179991 to 263899) | 0.61 (0.51 to 0.7) | 745.8 (610.88 to 890.03) | 535.53 (436.61 to 640.15) | -0.46 (-1.01 to 0.08) | 693.74 (575.35 to 827.1) | 505.87 (413.72 to 603.95) | -0.41 (-0.93 to 0.1) |
| Ireland | 16489 (11933 to 22087) | 21049 (14865 to 28876) | 0.28 (0.21 to 0.35) | 457.74 (331.29 to 613.17) | 425.97 (300.83 to 584.37) | -0.25 (-0.45 to -0.06) | 457.79 (332.43 to 612.94) | 456.39 (319.56 to 628.48) | -0.01 (-0.22 to 0.2) |
| Israel | 20098 (14553 to 26710) | 36081 (25836 to 48992) | 0.8 (0.71 to 0.87) | 405.11 (293.35 to 538.4) | 376.08 (269.29 to 510.66) | -0.21 (-0.46 to 0.04) | 394.4 (287.15 to 523.68) | 382.86 (274.73 to 519.78) | -0.06 (-0.31 to 0.2) |
| Italy | 350953 (246154 to 474149) | 274850 (192521 to 376198) | -0.22 (-0.27 to -0.17) | 617.89 (433.38 to 834.79) | 459.53 (321.88 to 628.97) | -1.07 (-1.11 to -1.03) | 616.48 (441.22 to 833) | 503.6 (356.31 to 698.43) | -0.71 (-0.76 to -0.67) |
| Jamaica | 8588 (6934 to 10592) | 9426 (7642 to 11566) | 0.1 (0.04 to 0.16) | 363.06 (293.11 to 447.75) | 336.66 (272.95 to 413.07) | -0.36 (-0.48 to -0.24) | 343.05 (276.93 to 423.71) | 340.38 (275.89 to 413.99) | -0.15 (-0.27 to -0.03) |
| Japan | 451832 (335966 to 593466) | 328507 (241264 to 437150) | -0.27 (-0.32 to -0.23) | 359.09 (267 to 471.65) | 257.25 (188.93 to 342.33) | -1.22 (-1.37 to -1.07) | 370.26 (274.03 to 486.46) | 274.93 (201.74 to 363.68) | -1.05 (-1.23 to -0.87) |
| Jordan | 17580 (14373 to 21483) | 45818 (36585 to 57103) | 1.61 (1.44 to 1.78) | 470.59 (384.74 to 575.08) | 371.76 (296.85 to 463.32) | -0.76 (-0.85 to -0.68) | 421.48 (348.54 to 509.37) | 349.36 (280.53 to 432.78) | -0.6 (-0.67 to -0.53) |
| Kazakhstan | 131031 (107304 to 158277) | 125678 (100773 to 154076) | -0.04 (-0.08 to 0) | 799.32 (654.57 to 965.52) | 663.02 (531.63 to 812.83) | -0.5 (-0.62 to -0.39) | 765.74 (627.45 to 923.37) | 667.57 (534.65 to 819.21) | -0.36 (-0.45 to -0.27) |
| Kenya | 51804 (42676 to 62591) | 100172 (82860 to 120298) | 0.93 (0.85 to 1.02) | 223.8 (184.37 to 270.41) | 200.09 (165.51 to 240.3) | -0.54 (-0.73 to -0.35) | 229.42 (191.29 to 272.6) | 215.64 (179.19 to 259.31) | -0.33 (-0.5 to -0.16) |
| Kiribati | 154 (128 to 186) | 220 (179 to 271) | 0.43 (0.35 to 0.51) | 206.76 (171.39 to 249.95) | 181.52 (147.53 to 223.29) | -0.48 (-0.68 to -0.28) | 199.59 (165.87 to 239.66) | 176.28 (144.92 to 216.13) | -0.47 (-0.65 to -0.3) |
| Kuwait | 12949 (9902 to 18065) | 21761 (17258 to 27318) | 0.68 (0.2 to 1.05) | 753.5 (576.16 to 1051.15) | 467.98 (371.14 to 587.47) | -0.99 (-1.32 to -0.66) | 681.77 (520.66 to 978.87) | 453.41 (359.76 to 575.24) | -0.8 (-1.13 to -0.47) |
| Kyrgyzstan | 33396 (27070 to 41135) | 32164 (26189 to 38802) | -0.04 (-0.08 to 0.02) | 748.14 (606.42 to 921.51) | 468.65 (381.6 to 565.37) | -1.73 (-1.81 to -1.65) | 695.42 (566.1 to 853.75) | 450.96 (367.83 to 541.12) | -1.59 (-1.67 to -1.52) |
| Lao People's Democratic Republic | 14279 (11756 to 17951) | 19361 (15998 to 23270) | 0.36 (0.13 to 0.53) | 342.46 (281.96 to 430.52) | 262.43 (216.85 to 315.42) | -0.51 (-0.61 to -0.41) | 331.99 (275.84 to 408.86) | 259.14 (215.17 to 312.86) | -0.53 (-0.61 to -0.44) |
| Latvia | 36574 (29380 to 44870) | 14396 (11464 to 17715) | -0.61 (-0.62 to -0.59) | 1375.94 (1105.29 to 1688.03) | 769.7 (612.96 to 947.18) | -2.41 (-2.62 to -2.19) | 1395.74 (1119.93 to 1712.33) | 830.08 (661.73 to 1028.78) | -2.17 (-2.35 to -2) |
| Lebanon | 17371 (13941 to 22363) | 20697 (16704 to 25680) | 0.19 (-0.06 to 0.43) | 580.59 (465.93 to 747.43) | 373.57 (301.5 to 463.49) | -1.08 (-1.37 to -0.79) | 588.94 (465.49 to 745.74) | 371.17 (296.57 to 460.25) | -1.27 (-1.59 to -0.94) |
| Lesotho | 3684 (3102 to 4387) | 5506 (4664 to 6556) | 0.49 (0.4 to 0.59) | 240.41 (202.43 to 286.26) | 293.75 (248.83 to 349.78) | 0.76 (0.64 to 0.89) | 235.59 (200.51 to 277.44) | 285.41 (243.14 to 337.42) | 0.74 (0.61 to 0.88) |
| Liberia | 14124 (8948 to 25833) | 10754 (8709 to 13274) | -0.24 (-0.58 to 0.23) | 574.01 (363.63 to 1049.87) | 196.97 (159.51 to 243.14) | -2.83 (-3.75 to -1.89) | 503.74 (325.08 to 901.39) | 199.27 (162.62 to 244.29) | -2.38 (-3.24 to -1.51) |
| Libya | 21199 (17205 to 25889) | 33403 (28014 to 40579) | 0.58 (0.46 to 0.73) | 502.94 (408.19 to 614.21) | 486.17 (407.75 to 590.63) | 0.63 (0.2 to 1.07) | 468.4 (385.08 to 565.48) | 504.68 (419.07 to 616.21) | 1.08 (0.62 to 1.54) |
| Lithuania | 45750 (36744 to 56311) | 22987 (18082 to 29104) | -0.5 (-0.52 to -0.48) | 1245.13 (1000.02 to 1532.54) | 842.53 (662.74 to 1066.75) | -1.49 (-1.69 to -1.29) | 1246.47 (1004 to 1538.76) | 871.41 (695.23 to 1102.8) | -1.36 (-1.54 to -1.19) |
| Luxembourg | 2231 (1642 to 2978) | 3228 (2275 to 4396) | 0.45 (0.37 to 0.52) | 585.3 (430.81 to 781.26) | 501.04 (353.07 to 682.37) | -0.48 (-0.53 to -0.42) | 596.36 (438.25 to 793.86) | 519.13 (366.45 to 710.15) | -0.46 (-0.51 to -0.4) |
| Madagascar | 27913 (23110 to 33806) | 53235 (43464 to 64909) | 0.91 (0.82 to 1) | 234.55 (194.19 to 284.07) | 186.41 (152.2 to 227.29) | -0.76 (-0.83 to -0.68) | 218.08 (180.2 to 259.59) | 182.72 (150.78 to 219.86) | -0.61 (-0.67 to -0.55) |
| Malawi | 22455 (18481 to 27177) | 38626 (30979 to 47863) | 0.72 (0.63 to 0.81) | 229 (188.47 to 277.16) | 198.62 (159.29 to 246.11) | -0.53 (-0.61 to -0.46) | 212.28 (174.57 to 253.18) | 195.87 (159.89 to 238.61) | -0.34 (-0.4 to -0.27) |
| Malaysia | 48717 (40194 to 58164) | 89549 (73575 to 108487) | 0.84 (0.74 to 0.94) | 275.75 (227.51 to 329.22) | 281.49 (231.28 to 341.02) | 0.01 (-0.02 to 0.04) | 277.32 (230 to 330.63) | 276.73 (226.29 to 336.48) | -0.08 (-0.11 to -0.05) |
| Maldives | 755 (601 to 910) | 1594 (1273 to 1992) | 1.11 (0.89 to 1.3) | 339.7 (270.61 to 409.27) | 308.19 (246.06 to 385.13) | -0.22 (-0.74 to 0.31) | 336.39 (273.03 to 405.76) | 304.2 (242 to 382.15) | -0.3 (-0.81 to 0.22) |
| Mali | 24745 (20508 to 29848) | 63453 (52215 to 77229) | 1.56 (1.41 to 1.71) | 285.65 (236.73 to 344.55) | 263.25 (216.62 to 320.4) | -0.51 (-1.04 to 0.02) | 269.62 (224.98 to 324.52) | 255.92 (212.16 to 309.12) | -0.4 (-0.88 to 0.08) |
| Malta | 1823 (1299 to 2475) | 1937 (1377 to 2643) | 0.06 (0.01 to 0.12) | 491.88 (350.58 to 667.72) | 438.11 (311.46 to 597.65) | -0.3 (-0.5 to -0.11) | 510.76 (363.57 to 699.96) | 491.93 (346.21 to 676.38) | 0.05 (-0.21 to 0.32) |
| Marshall Islands | 123 (101 to 151) | 155 (128 to 192) | 0.26 (0.18 to 0.33) | 270.5 (222.12 to 331.54) | 275.67 (227.28 to 340.67) | 0.01 (-0.08 to 0.11) | 273.75 (229.89 to 330.49) | 271.76 (224.35 to 336.98) | -0.05 (-0.12 to 0.02) |
| Mauritania | 5140 (4302 to 6208) | 9559 (7860 to 11771) | 0.86 (0.75 to 0.98) | 250.17 (209.4 to 302.17) | 217.44 (178.79 to 267.75) | -0.5 (-0.56 to -0.44) | 248.21 (208.29 to 294.92) | 218.76 (182.76 to 267.16) | -0.47 (-0.51 to -0.42) |
| Mauritius | 2870 (2355 to 3478) | 3091 (2491 to 3814) | 0.08 (0.01 to 0.15) | 261.9 (214.87 to 317.37) | 243.01 (195.81 to 299.88) | 0.03 (-0.07 to 0.13) | 248.92 (204.89 to 299.28) | 247.76 (200.04 to 308.96) | 0.24 (0.14 to 0.35) |
| Mexico | 759958 (590925 to 957995) | 671488 (530926 to 837401) | -0.12 (-0.15 to -0.08) | 889.97 (692.02 to 1121.89) | 519.42 (410.69 to 647.76) | -0.52 (-1.03 to 0) | 826.97 (654.81 to 1030.35) | 522.65 (411.79 to 652.51) | -0.3 (-0.81 to 0.2) |
| Mongolia | 14396 (11895 to 17250) | 24211 (19663 to 29722) | 0.68 (0.58 to 0.78) | 667.14 (551.24 to 799.4) | 725.68 (589.36 to 890.86) | 0.44 (0.31 to 0.56) | 617.3 (513.12 to 733.1) | 710.16 (575.27 to 872.29) | 0.63 (0.54 to 0.71) |
| Montenegro | 6676 (5309 to 8228) | 5198 (4102 to 6392) | -0.22 (-0.25 to -0.19) | 1066.05 (847.8 to 1313.96) | 841 (663.64 to 1034.24) | -0.76 (-0.83 to -0.69) | 1062.17 (844.83 to 1310.13) | 905.6 (709.8 to 1130.08) | -0.51 (-0.58 to -0.43) |
| Morocco | 148635 (121746 to 179013) | 175255 (141315 to 214284) | 0.18 (0.1 to 0.25) | 586.17 (480.13 to 705.97) | 471.41 (380.12 to 576.39) | -0.85 (-0.96 to -0.74) | 550.16 (453.47 to 659.2) | 468.89 (379.41 to 572.92) | -0.63 (-0.73 to -0.54) |
| Mozambique | 38001 (30279 to 50526) | 72460 (58764 to 88578) | 0.91 (0.62 to 1.11) | 284.44 (226.64 to 378.19) | 233.2 (189.12 to 285.07) | -0.43 (-0.6 to -0.27) | 262.83 (210.64 to 337.33) | 232.1 (192.87 to 278.56) | -0.18 (-0.33 to -0.03) |
| Myanmar | 167733 (138978 to 200751) | 227623 (184111 to 280946) | 0.36 (0.23 to 0.52) | 414.79 (343.68 to 496.44) | 403.45 (326.32 to 497.96) | -0.32 (-1.31 to 0.69) | 397.21 (330.22 to 475.06) | 401.21 (324.89 to 496.32) | -0.15 (-1.15 to 0.86) |
| Namibia | 3366 (2819 to 4001) | 5932 (4970 to 7093) | 0.76 (0.69 to 0.83) | 239.75 (200.8 to 284.95) | 244.02 (204.42 to 291.75) | -0.04 (-0.19 to 0.12) | 237.5 (200.95 to 280.53) | 238.66 (200.82 to 282.7) | -0.11 (-0.25 to 0.03) |
| Nepal | 93371 (72274 to 122451) | 166948 (127146 to 219040) | 0.79 (0.68 to 0.9) | 479.58 (371.22 to 628.95) | 536.3 (408.44 to 703.64) | 0.29 (-0.13 to 0.71) | 474.35 (369.14 to 614.19) | 521.49 (399.03 to 683.16) | 0.26 (-0.13 to 0.66) |
| Netherlands | 51837 (37555 to 69255) | 74678 (53144 to 102032) | 0.44 (0.32 to 0.57) | 347.39 (251.67 to 464.12) | 433.91 (308.79 to 592.84) | 1.07 (0.54 to 1.6) | 353.12 (254.44 to 472.88) | 383.24 (270.05 to 519.77) | 0.51 (0.11 to 0.91) |
| New Zealand | 23887 (17910 to 30966) | 31534 (23181 to 41551) | 0.32 (0.24 to 0.42) | 699 (524.11 to 906.16) | 610.01 (448.43 to 803.76) | -0.44 (-0.62 to -0.27) | 706.73 (524.84 to 916.15) | 665.53 (477.96 to 889.75) | -0.22 (-0.47 to 0.02) |
| Nicaragua | 20904 (16163 to 26419) | 26719 (20917 to 33795) | 0.28 (0.22 to 0.35) | 537.71 (415.77 to 679.59) | 400.64 (313.65 to 506.75) | -1.1 (-1.45 to -0.74) | 451.15 (358.67 to 553.52) | 400.39 (312.69 to 504) | -0.48 (-0.82 to -0.13) |
| Niger | 21521 (17811 to 25575) | 63614 (52114 to 78442) | 1.96 (1.81 to 2.15) | 267.93 (221.74 to 318.41) | 254.07 (208.14 to 313.29) | -0.12 (-0.2 to -0.05) | 249.85 (209.92 to 297.57) | 246.74 (204.76 to 300.23) | 0.02 (-0.04 to 0.08) |
| Nigeria | 222977 (182690 to 270432) | 500436 (408101 to 614922) | 1.24 (1.15 to 1.34) | 247.64 (202.89 to 300.34) | 216.48 (176.54 to 266.01) | -0.46 (-0.56 to -0.36) | 237.37 (195.62 to 285.93) | 217.61 (178.42 to 264.38) | -0.3 (-0.38 to -0.22) |
| Northern Mariana Islands | 193 (158 to 236) | 183 (147 to 231) | -0.05 (-0.11 to 0.02) | 427.02 (351.19 to 521.99) | 378.33 (303.28 to 475.64) | -0.62 (-0.74 to -0.49) | 401.68 (328.24 to 491.71) | 390.15 (312.41 to 493.88) | -0.15 (-0.24 to -0.05) |
| Norway | 22474 (16299 to 30061) | 24477 (17472 to 33643) | 0.09 (0.03 to 0.14) | 529.28 (383.86 to 707.96) | 451.76 (322.48 to 620.94) | -0.54 (-0.7 to -0.38) | 505.48 (365.82 to 673.31) | 423.22 (296.9 to 581.3) | -0.55 (-0.74 to -0.36) |
| Oman | 13525 (11056 to 16280) | 25287 (20698 to 31122) | 0.87 (0.71 to 1.03) | 681.56 (557.14 to 820.42) | 537.6 (440.04 to 661.65) | -0.72 (-0.91 to -0.54) | 666.68 (547.99 to 801.01) | 541.43 (441.97 to 670.9) | -0.8 (-0.92 to -0.67) |
| Pakistan | 283456 (229282 to 354565) | 515491 (427343 to 629560) | 0.82 (0.73 to 0.9) | 255.07 (206.32 to 319.06) | 218.84 (181.42 to 267.27) | -0.36 (-0.76 to 0.04) | 245.26 (201.87 to 300.62) | 212.95 (178.7 to 256.53) | -0.36 (-0.73 to 0.01) |
| Palestine | 11221 (8949 to 14238) | 23507 (18860 to 29435) | 1.1 (0.82 to 1.31) | 548.17 (437.21 to 695.59) | 457.74 (367.25 to 573.15) | -0.52 (-1.36 to 0.32) | 490.14 (394.26 to 615.91) | 422.82 (341.25 to 526.56) | -0.43 (-1.2 to 0.34) |
| Panama | 12625 (9946 to 15877) | 17892 (14126 to 22402) | 0.42 (0.37 to 0.47) | 528.49 (416.34 to 664.59) | 416.87 (329.11 to 521.94) | -0.85 (-0.92 to -0.78) | 489.66 (388.79 to 612.65) | 421.12 (331.28 to 528.92) | -0.56 (-0.63 to -0.5) |
| Papua New Guinea | 12320 (9916 to 15400) | 38238 (30689 to 48474) | 2.1 (1.9 to 2.28) | 300.24 (241.67 to 375.32) | 365.5 (293.35 to 463.36) | 0.12 (-0.49 to 0.74) | 303.89 (247.14 to 377.96) | 376.74 (302.31 to 475.49) | 0.26 (-0.27 to 0.8) |
| Paraguay | 24721 (19130 to 31378) | 37271 (29740 to 46161) | 0.51 (0.42 to 0.6) | 611.49 (473.21 to 776.17) | 519.9 (414.85 to 643.92) | -0.56 (-0.68 to -0.45) | 562.7 (446.42 to 711.14) | 503.2 (402.02 to 622.79) | -0.4 (-0.49 to -0.3) |
| Peru | 99321 (82264 to 121862) | 139327 (112293 to 169096) | 0.4 (0.18 to 0.57) | 459.09 (380.25 to 563.28) | 384.13 (309.6 to 466.2) | -0.39 (-0.49 to -0.29) | 424.79 (351.8 to 515.58) | 379.19 (305.41 to 460.97) | -0.2 (-0.29 to -0.11) |
| Philippines | 239888 (198839 to 293439) | 297447 (242841 to 362957) | 0.24 (0.13 to 0.33) | 380.72 (315.57 to 465.71) | 262.65 (214.43 to 320.49) | -0.86 (-1.14 to -0.59) | 364.88 (304.72 to 440.33) | 259.05 (212.09 to 316.18) | -0.8 (-1.05 to -0.55) |
| Poland | 418012 (337865 to 507993) | 316484 (249898 to 389255) | -0.24 (-0.28 to -0.21) | 1095.06 (885.1 to 1330.78) | 827.66 (653.53 to 1017.97) | -1.01 (-1.12 to -0.89) | 1100.06 (882.68 to 1340.63) | 854.44 (676.77 to 1056.12) | -0.88 (-0.98 to -0.78) |
| Portugal | 48533 (36466 to 63371) | 32806 (23970 to 43653) | -0.32 (-0.37 to -0.28) | 478.77 (359.73 to 625.14) | 309.26 (225.97 to 411.52) | -1.63 (-1.72 to -1.54) | 493.1 (371.39 to 644.6) | 318.9 (231.14 to 426.55) | -1.62 (-1.7 to -1.54) |
| Puerto Rico | 14732 (12159 to 17693) | 13955 (11197 to 17314) | -0.05 (-0.12 to 0.02) | 407.82 (336.61 to 489.79) | 423.6 (339.88 to 525.58) | 0.21 (0.04 to 0.38) | 406.61 (335.84 to 489.12) | 442.63 (356.1 to 548.98) | 0.38 (0.19 to 0.58) |
| Qatar | 2894 (2378 to 3488) | 17326 (13910 to 21593) | 4.99 (4.57 to 5.42) | 650.55 (534.59 to 784.03) | 582 (467.23 to 725.32) | 0.04 (-0.14 to 0.21) | 569.24 (465.18 to 684.06) | 523.65 (419.21 to 657.9) | -0.2 (-0.31 to -0.08) |
| Republic of Korea | 266471 (201812 to 348215) | 213144 (155280 to 284795) | -0.2 (-0.26 to -0.13) | 602.26 (456.12 to 787.01) | 413.31 (301.1 to 552.24) | -1.48 (-1.56 to -1.41) | 575.36 (435.17 to 747.73) | 406.59 (293.52 to 541.79) | -1.39 (-1.49 to -1.29) |
| Republic of Moldova | 46857 (38213 to 56860) | 21887 (17735 to 26933) | -0.53 (-0.55 to -0.51) | 1053.58 (859.24 to 1278.51) | 609.07 (493.54 to 749.5) | -1.95 (-2.02 to -1.88) | 1052.57 (857.21 to 1279.62) | 661.78 (533.16 to 818.18) | -1.69 (-1.76 to -1.62) |
| Romania | 291568 (233435 to 358953) | 160260 (126869 to 196512) | -0.45 (-0.47 to -0.43) | 1247.09 (998.45 to 1535.31) | 846.21 (669.9 to 1037.63) | -1.48 (-1.56 to -1.41) | 1286.02 (1029.55 to 1586.31) | 944.84 (746.72 to 1163.53) | -1.18 (-1.24 to -1.12) |
| Russian Federation | 1726495 (1427277 to 2070674) | 1288347 (1039715 to 1588734) | -0.25 (-0.29 to -0.21) | 1143.49 (945.31 to 1371.44) | 889.41 (717.77 to 1096.78) | -1.1 (-1.52 to -0.68) | 1167.73 (952.27 to 1400.75) | 943.03 (754.92 to 1171.43) | -0.98 (-1.32 to -0.64) |
| Rwanda | 26177 (20012 to 38462) | 27012 (22015 to 32740) | 0.03 (-0.29 to 0.33) | 364.11 (278.35 to 534.98) | 203.56 (165.9 to 246.72) | -2.72 (-4.03 to -1.4) | 335.96 (262.04 to 467.67) | 205.19 (169.58 to 247.2) | -2.46 (-3.75 to -1.15) |
| Saint Kitts and Nevis | 153 (126 to 187) | 220 (179 to 270) | 0.43 (0.35 to 0.52) | 370.08 (304.79 to 449.93) | 374.88 (305.1 to 460.46) | 0.12 (-0.08 to 0.32) | 363.19 (299.21 to 440.81) | 395.92 (321.65 to 480.72) | 0.38 (0.22 to 0.54) |
| Saint Lucia | 449 (371 to 540) | 558 (459 to 676) | 0.24 (0.16 to 0.33) | 328.96 (271.63 to 395.75) | 314.08 (258.74 to 380.96) | -0.19 (-0.36 to -0.03) | 311.27 (259.83 to 372.45) | 332.95 (273.21 to 402.85) | 0.19 (0.04 to 0.34) |
| Saint Vincent and the Grenadines | 370 (303 to 446) | 394 (322 to 478) | 0.06 (0 to 0.12) | 337.94 (276.51 to 407.12) | 345.39 (282.15 to 419.23) | -0.01 (-0.17 to 0.16) | 316.96 (262.81 to 378.9) | 353.53 (287.42 to 429.98) | 0.3 (0.14 to 0.46) |
| Samoa | 518 (425 to 644) | 585 (471 to 733) | 0.13 (-0.03 to 0.25) | 306.62 (251.42 to 381.2) | 273.59 (220.69 to 342.96) | 0.01 (-0.87 to 0.9) | 295.58 (242.59 to 360.19) | 279.03 (227.06 to 348.47) | 0.14 (-0.67 to 0.96) |
| Sao Tome and Principe | 373 (297 to 467) | 722 (570 to 908) | 0.94 (0.78 to 1.16) | 307.5 (245.11 to 384.8) | 333.46 (262.94 to 419.38) | 0.03 (-0.09 to 0.15) | 275.1 (222.25 to 335.98) | 333.79 (265.14 to 421) | 0.41 (0.33 to 0.49) |
| Saudi Arabia | 158477 (126687 to 197792) | 464989 (364227 to 586395) | 1.93 (1.71 to 2.15) | 999.45 (798.96 to 1247.39) | 1233.26 (966.01 to 1555.25) | 0.89 (0.77 to 1) | 971.57 (781.84 to 1200.72) | 1068.92 (842.57 to 1346.95) | 0.4 (0.34 to 0.46) |
| Senegal | 17590 (14375 to 21628) | 32869 (26500 to 40595) | 0.87 (0.76 to 0.99) | 230.46 (188.34 to 283.37) | 207.25 (167.09 to 255.96) | -0.42 (-0.5 to -0.34) | 214.94 (177.09 to 258.08) | 208.82 (169.87 to 255.4) | -0.16 (-0.23 to -0.09) |
| Serbia | 85458 (68604 to 103298) | 66176 (52532 to 81564) | -0.23 (-0.27 to -0.18) | 887.45 (712.43 to 1072.72) | 741.93 (588.96 to 914.45) | -0.7 (-0.88 to -0.51) | 926.39 (740.37 to 1122.28) | 809.01 (633.56 to 1001.88) | -0.52 (-0.72 to -0.32) |
| Seychelles | 218 (180 to 262) | 274 (224 to 337) | 0.26 (0.19 to 0.33) | 298.86 (246.62 to 359.46) | 259.83 (212.25 to 319.18) | -0.5 (-0.62 to -0.39) | 292.99 (242.7 to 350.36) | 260.69 (213.05 to 323.05) | -0.42 (-0.55 to -0.29) |
| Sierra Leone | 10065 (8361 to 12095) | 18953 (15473 to 23342) | 0.88 (0.79 to 0.97) | 242.43 (201.38 to 291.32) | 213.74 (174.49 to 263.24) | -1.8 (-2.58 to -1.01) | 219.33 (182.44 to 260.38) | 209.7 (170.98 to 255.74) | -1.52 (-2.25 to -0.78) |
| Singapore | 11529 (8665 to 15093) | 16245 (11875 to 21710) | 0.41 (0.34 to 0.5) | 378.36 (284.36 to 495.32) | 283.65 (207.33 to 379.05) | -1.04 (-1.26 to -0.83) | 357.8 (266.23 to 466.28) | 327.9 (238.93 to 436.65) | -0.43 (-0.6 to -0.27) |
| Slovakia | 66828 (52936 to 82261) | 55319 (43412 to 68523) | -0.17 (-0.21 to -0.14) | 1264.9 (1001.96 to 1557.01) | 1018.87 (799.56 to 1262.06) | -0.71 (-0.74 to -0.68) | 1257.22 (994.95 to 1549.6) | 1059.01 (825.72 to 1314.3) | -0.53 (-0.57 to -0.49) |
| Slovenia | 29583 (23358 to 36478) | 24347 (19030 to 30606) | -0.18 (-0.22 to -0.13) | 1498.99 (1183.55 to 1848.35) | 1176.39 (919.49 to 1478.8) | -0.42 (-0.65 to -0.19) | 1486.56 (1171.09 to 1831.21) | 1172.8 (916.72 to 1469.73) | -0.37 (-0.64 to -0.1) |
| Solomon Islands | 1276 (1030 to 1603) | 3238 (2539 to 4138) | 1.54 (1.4 to 1.69) | 376.37 (303.85 to 472.84) | 473.72 (371.42 to 605.38) | 0.81 (0.65 to 0.96) | 417.36 (338.47 to 518.13) | 506.01 (394.49 to 640.21) | 0.69 (0.57 to 0.8) |
| Somalia | 27026 (20477 to 40660) | 55790 (45855 to 68376) | 1.06 (0.68 to 1.4) | 340.43 (257.94 to 512.18) | 258.21 (212.23 to 316.46) | -0.16 (-0.74 to 0.43) | 321.82 (249.99 to 459.76) | 257.41 (216.16 to 308.72) | -0.09 (-0.58 to 0.42) |
| South Africa | 146520 (122664 to 174993) | 150459 (126586 to 179272) | 0.03 (-0.01 to 0.06) | 395.86 (331.41 to 472.79) | 264.64 (222.65 to 315.32) | -1.44 (-1.59 to -1.29) | 381.98 (320.26 to 457.01) | 254.86 (213.49 to 304.27) | -1.47 (-1.61 to -1.34) |
| South Sudan | 13601 (11313 to 16415) | 22857 (19016 to 27992) | 0.68 (0.55 to 0.93) | 231.42 (192.49 to 279.29) | 236.31 (196.6 to 289.4) | 0.09 (-0.63 to 0.82) | 218.77 (183.59 to 259.26) | 227.36 (191.2 to 272.73) | 0.16 (-0.47 to 0.8) |
| Spain | 159194 (118348 to 209103) | 178959 (127682 to 242957) | 0.12 (0.03 to 0.21) | 410.48 (305.16 to 539.17) | 392.89 (280.32 to 533.39) | -0.15 (-0.28 to -0.01) | 417.82 (312.31 to 549.59) | 443.16 (314.44 to 609.87) | 0.24 (0.09 to 0.4) |
| Sri Lanka | 99869 (75682 to 144887) | 91461 (72910 to 114230) | -0.08 (-0.38 to 0.21) | 582.99 (441.79 to 845.78) | 410.69 (327.39 to 512.93) | -1.14 (-1.92 to -0.36) | 546.62 (418.78 to 768.33) | 405.67 (324.11 to 506.09) | -0.93 (-1.74 to -0.12) |
| Sudan | 110281 (88839 to 140032) | 177761 (144944 to 217670) | 0.61 (0.32 to 0.85) | 550.82 (443.72 to 699.41) | 409.41 (333.83 to 501.32) | -0.81 (-1.02 to -0.6) | 501.41 (408.48 to 626.05) | 381.32 (312.71 to 463.94) | -0.75 (-0.94 to -0.55) |
| Suriname | 1185 (987 to 1411) | 1772 (1466 to 2104) | 0.49 (0.43 to 0.56) | 306.49 (255.21 to 364.76) | 305.83 (253.08 to 363.19) | 0.04 (-0.07 to 0.14) | 290.03 (243.66 to 344.86) | 310.7 (256.87 to 368.47) | 0.27 (0.17 to 0.38) |
| Sweden | 40760 (29014 to 54767) | 43325 (30549 to 59617) | 0.06 (0.01 to 0.11) | 474.63 (337.85 to 637.73) | 417.65 (294.49 to 574.7) | -0.39 (-0.56 to -0.23) | 477.01 (339.71 to 641.69) | 400.9 (282.21 to 552.56) | -0.51 (-0.71 to -0.31) |
| Switzerland | 52389 (38076 to 71002) | 51601 (36443 to 70783) | -0.02 (-0.06 to 0.03) | 762.97 (554.53 to 1034.04) | 578.3 (408.42 to 793.28) | -1.2 (-1.35 to -1.06) | 753.76 (540.02 to 1014.04) | 578.23 (408.23 to 794.54) | -1.23 (-1.47 to -0.99) |
| Syrian Arab Republic | 57748 (45880 to 73106) | 64240 (50712 to 82954) | 0.11 (0.02 to 0.23) | 454.08 (360.76 to 574.84) | 457.86 (361.44 to 591.25) | 2.23 (1.34 to 3.13) | 405.1 (325.83 to 502.64) | 452.18 (359.25 to 577.93) | 2.61 (1.7 to 3.54) |
| Taiwan (Province of China) | 87431 (71131 to 108787) | 55783 (44324 to 70221) | -0.36 (-0.4 to -0.32) | 428.78 (348.84 to 533.51) | 236 (187.52 to 297.08) | -2.47 (-2.72 to -2.22) | 417.55 (339.38 to 520.78) | 218.47 (175.54 to 271.94) | -2.63 (-2.9 to -2.36) |
| Tajikistan | 35468 (28775 to 43529) | 45870 (36926 to 55980) | 0.29 (0.24 to 0.35) | 660.61 (535.95 to 810.74) | 451.48 (363.45 to 550.99) | -2.19 (-2.69 to -1.69) | 595.85 (486.88 to 723.76) | 425.09 (343.49 to 517.06) | -2 (-2.48 to -1.51) |
| Thailand | 231830 (194327 to 271558) | 239414 (195507 to 293494) | 0.03 (-0.06 to 0.12) | 408.41 (342.34 to 478.39) | 359.03 (293.19 to 440.13) | -0.67 (-0.81 to -0.53) | 388.43 (326.98 to 456.35) | 359.91 (296.44 to 440.04) | -0.47 (-0.63 to -0.3) |
| Timor-Leste | 3334 (2476 to 5175) | 3599 (2986 to 4379) | 0.08 (-0.3 to 0.47) | 426.87 (316.98 to 662.48) | 257.5 (213.65 to 313.32) | -2.88 (-3.86 to -1.89) | 395.93 (300.99 to 594.82) | 252.99 (209.99 to 305.39) | -2.58 (-3.49 to -1.67) |
| Togo | 9017 (7468 to 10912) | 18365 (15159 to 22513) | 1.04 (0.95 to 1.12) | 247.27 (204.79 to 299.22) | 219.4 (181.09 to 268.94) | -0.47 (-0.56 to -0.38) | 236.75 (198.45 to 282.61) | 224.1 (186.69 to 273.77) | -0.25 (-0.33 to -0.17) |
| Tokelau | 4 (3 to 5) | 4 (3 to 5) | -0.12 (-0.17 to -0.06) | 253.46 (205.74 to 312.88) | 260.39 (207.82 to 329.04) | 0.05 (-0.04 to 0.14) | 255.59 (209.89 to 312.24) | 258.5 (207.07 to 324.82) | 0 (-0.08 to 0.08) |
| Tonga | 252 (206 to 313) | 234 (187 to 294) | -0.07 (-0.13 to -0.02) | 254.72 (208.54 to 316.98) | 219.91 (175.76 to 276.81) | -0.38 (-0.68 to -0.08) | 252.95 (209.56 to 310.14) | 221.08 (178 to 277.92) | -0.35 (-0.6 to -0.09) |
| Trinidad and Tobago | 4137 (3410 to 4967) | 4214 (3513 to 5030) | 0.02 (-0.02 to 0.07) | 343.37 (283.07 to 412.24) | 302.51 (252.14 to 361.06) | -0.2 (-0.34 to -0.06) | 329.29 (272.81 to 391.72) | 327.05 (270.26 to 392.22) | 0.22 (0.09 to 0.34) |
| Tunisia | 44184 (35801 to 53491) | 51061 (41339 to 62048) | 0.16 (0.09 to 0.22) | 529.18 (428.78 to 640.65) | 431.16 (349.07 to 523.94) | -0.67 (-0.74 to -0.59) | 493.61 (401.64 to 594.89) | 441.46 (356.68 to 537.82) | -0.37 (-0.42 to -0.32) |
| Turkmenistan | 22807 (18459 to 27825) | 22961 (18489 to 27724) | 0.01 (-0.05 to 0.08) | 616.53 (499 to 752.19) | 445.12 (358.43 to 537.46) | -1.1 (-1.21 to -0.99) | 540.66 (440.95 to 651.04) | 431.82 (347.97 to 521.6) | -0.76 (-0.88 to -0.64) |
| Tuvalu | 26 (22 to 31) | 35 (28 to 43) | 0.32 (0.24 to 0.4) | 275.77 (231.59 to 330.69) | 279.4 (230.21 to 347.54) | -0.34 (-0.51 to -0.17) | 278.95 (235.13 to 333.43) | 277.95 (229.39 to 344.67) | -0.44 (-0.63 to -0.25) |
| Uganda | 41085 (33689 to 49934) | 91385 (74435 to 112761) | 1.22 (1 to 1.38) | 237.61 (194.83 to 288.79) | 210.98 (171.85 to 260.33) | -0.5 (-0.69 to -0.3) | 221.65 (184.54 to 266.79) | 207.79 (172.44 to 251.84) | -0.33 (-0.5 to -0.17) |
| Ukraine | 617351 (491035 to 777915) | 384062 (308480 to 477781) | -0.38 (-0.41 to -0.34) | 1171.15 (931.52 to 1475.75) | 891.55 (716.1 to 1109.11) | -1.35 (-1.55 to -1.15) | 1222.73 (968.85 to 1543.43) | 969.5 (776.56 to 1205.13) | -1.18 (-1.35 to -1.02) |
| United Arab Emirates | 11816 (9680 to 14189) | 55269 (44702 to 68141) | 3.68 (3.39 to 4) | 631.56 (517.41 to 758.43) | 573.85 (464.14 to 707.5) | 0.02 (-0.15 to 0.19) | 581.14 (475.35 to 703.54) | 547.56 (440.98 to 679.72) | -0.22 (-0.24 to -0.19) |
| United Republic of Tanzania | 63265 (52161 to 76287) | 129372 (105569 to 158979) | 1.04 (0.95 to 1.14) | 244.86 (201.88 to 295.26) | 221.35 (180.62 to 272) | -0.31 (-0.35 to -0.27) | 228.11 (190.69 to 272.9) | 216.6 (178.73 to 264.42) | -0.18 (-0.21 to -0.15) |
| United States of America | 941217 (706485 to 1229537) | 1202247 (899155 to 1618912) | 0.28 (0.16 to 0.41) | 370.42 (278.04 to 483.89) | 361.42 (270.31 to 486.68) | -0.15 (-0.38 to 0.08) | 366.93 (275.49 to 478.07) | 308.14 (229.8 to 404.42) | -0.71 (-0.95 to -0.48) |
| United States Virgin Islands | 369 (310 to 437) | 271 (225 to 326) | -0.27 (-0.31 to -0.22) | 347.98 (292.06 to 411.99) | 315.73 (261.52 to 379.02) | -0.53 (-0.68 to -0.38) | 347.87 (292.68 to 410.98) | 327.07 (271.89 to 393.28) | -0.43 (-0.63 to -0.22) |
| Uruguay | 12972 (9709 to 17183) | 13043 (9740 to 16829) | 0.01 (-0.08 to 0.08) | 413.21 (309.27 to 547.33) | 382.98 (285.99 to 494.16) | -0.37 (-0.42 to -0.31) | 419.13 (314.72 to 556.11) | 393.83 (291.49 to 509.04) | -0.31 (-0.37 to -0.26) |
| Uzbekistan | 136181 (109861 to 166003) | 162567 (132818 to 196061) | 0.19 (0.14 to 0.25) | 649.75 (524.17 to 792.04) | 474.88 (387.98 to 572.73) | -1.15 (-1.32 to -0.99) | 582 (475.53 to 703.73) | 467.86 (381.87 to 565.77) | -0.84 (-0.97 to -0.7) |
| Vanuatu | 376 (305 to 463) | 756 (619 to 938) | 1.01 (0.9 to 1.11) | 246.88 (200.14 to 304.12) | 241.52 (197.89 to 299.57) | -0.23 (-0.63 to 0.18) | 245.21 (201.33 to 299.85) | 241.15 (198.41 to 297.87) | -0.18 (-0.51 to 0.15) |
| Yemen | 64797 (53783 to 78072) | 196477 (152374 to 285054) | 2.03 (1.39 to 3.46) | 475.25 (394.46 to 572.61) | 584 (452.91 to 847.28) | 0.94 (0.6 to 1.29) | 440.48 (370.34 to 525.69) | 537.5 (423.11 to 762.21) | 0.89 (0.57 to 1.22) |
| Zambia | 18954 (15686 to 22930) | 42399 (34592 to 52537) | 1.24 (1.12 to 1.36) | 238.82 (197.64 to 288.92) | 217.26 (177.26 to 269.21) | -0.36 (-0.43 to -0.3) | 226.06 (189.12 to 269.3) | 225.97 (187.03 to 277.13) | -0.04 (-0.1 to 0.02) |
| Zimbabwe | 23280 (19261 to 27973) | 32670 (27367 to 39306) | 0.4 (0.35 to 0.46) | 225.09 (186.23 to 270.46) | 209.49 (175.48 to 252.03) | -0.29 (-0.4 to -0.18) | 227.61 (190.28 to 269.63) | 213.23 (180.58 to 251.93) | -0.26 (-0.35 to -0.17) |

CR, crude rate; ASR, age-standardized rate; EAPC, estimated annual percentage change; UI, uncertainty interval; CI, confidence interval.

Table S4. YLDs of forearm fracture in 1990 and 2021 for both sexes in countries and territories, with EAPC from 1990 to 2021.

| **Location** | **Number in 1990 (95% UI)** | **Number in 2021 (95% UI)** | **Number change rate (95% UI)** | **CR in 1990 (95% UI)** | **CR in 2021 (95% UI)** | **EAPC of CR, % per year (95% CI)** | **ASR in 1990 (95% UI)** | **ASR in 2021 (95% UI)** | **EAPC of ASR, % per year (95% CI)** |
| --- | --- | --- | --- | --- | --- | --- | --- | --- | --- |
| Afghanistan | 299 (179 to 518) | 1000 (553 to 1772) | 2.35 (1.47 to 3.72) | 3 (1.8 to 5.21) | 3.2 (1.77 to 5.67) | -0.17 (-0.53 to 0.19) | 3.39 (2.03 to 5.86) | 3.64 (2.11 to 6.22) | -0.08 (-0.3 to 0.14) |
| Albania | 166 (97 to 268) | 134 (81 to 214) | -0.19 (-0.26 to -0.1) | 5.02 (2.94 to 8.12) | 5.03 (3.05 to 8.01) | -0.24 (-0.41 to -0.07) | 5.24 (3.12 to 8.41) | 4.71 (2.81 to 7.64) | -0.61 (-0.78 to -0.44) |
| Algeria | 721 (423 to 1149) | 1031 (616 to 1598) | 0.43 (0.32 to 0.56) | 2.85 (1.67 to 4.54) | 2.33 (1.39 to 3.62) | -0.81 (-0.88 to -0.73) | 3.24 (1.95 to 5.07) | 2.42 (1.45 to 3.74) | -1.1 (-1.15 to -1.04) |
| American Samoa | 1 (0 to 1) | 1 (1 to 1) | 0.17 (0.06 to 0.3) | 1.51 (0.88 to 2.4) | 1.72 (1.06 to 2.67) | 0.49 (-0.02 to 1) | 1.82 (1.09 to 2.84) | 1.77 (1.1 to 2.74) | 0.02 (-0.43 to 0.48) |
| Andorra | 2 (1 to 3) | 5 (3 to 7) | 1.25 (1.05 to 1.49) | 3.92 (2.34 to 6.1) | 5.6 (3.45 to 8.54) | 1.12 (1.02 to 1.21) | 3.86 (2.3 to 6.06) | 4.11 (2.49 to 6.52) | 0.21 (0.13 to 0.29) |
| Angola | 165 (98 to 281) | 360 (220 to 584) | 1.17 (0.77 to 1.52) | 1.61 (0.95 to 2.74) | 1.1 (0.67 to 1.79) | -1.58 (-1.99 to -1.17) | 1.89 (1.15 to 3.03) | 1.52 (0.94 to 2.34) | -0.96 (-1.25 to -0.67) |
| Antigua and Barbuda | 1 (1 to 2) | 2 (1 to 3) | 0.64 (0.51 to 0.8) | 1.96 (1.17 to 3.12) | 2.17 (1.32 to 3.42) | 0.2 (-0.04 to 0.44) | 1.98 (1.2 to 3.15) | 2.1 (1.27 to 3.34) | 0.05 (-0.2 to 0.31) |
| Argentina | 642 (379 to 1025) | 901 (542 to 1471) | 0.4 (0.28 to 0.54) | 1.94 (1.15 to 3.1) | 1.98 (1.19 to 3.23) | 0.07 (-0.11 to 0.25) | 1.97 (1.17 to 3.14) | 1.88 (1.13 to 3.07) | -0.12 (-0.3 to 0.07) |
| Armenia | 144 (88 to 228) | 76 (47 to 118) | -0.47 (-0.52 to -0.41) | 4.21 (2.56 to 6.67) | 2.53 (1.55 to 3.95) | -1.63 (-1.96 to -1.29) | 4.36 (2.7 to 6.87) | 2.35 (1.41 to 3.75) | -1.95 (-2.35 to -1.55) |
| Australia | 588 (357 to 935) | 1011 (618 to 1573) | 0.72 (0.58 to 0.88) | 3.48 (2.12 to 5.54) | 3.92 (2.4 to 6.1) | 0.48 (0.38 to 0.59) | 3.42 (2.06 to 5.45) | 3.29 (1.94 to 5.26) | -0.04 (-0.17 to 0.08) |
| Austria | 350 (213 to 552) | 360 (223 to 557) | 0.03 (-0.05 to 0.13) | 4.5 (2.74 to 7.1) | 4.01 (2.48 to 6.2) | -0.18 (-0.27 to -0.09) | 3.85 (2.3 to 6.12) | 3.03 (1.81 to 4.75) | -0.64 (-0.74 to -0.55) |
| Azerbaijan | 202 (118 to 324) | 244 (149 to 385) | 0.21 (0.11 to 0.35) | 2.75 (1.61 to 4.42) | 2.33 (1.42 to 3.67) | -0.68 (-0.88 to -0.49) | 2.84 (1.68 to 4.52) | 2.3 (1.38 to 3.67) | -0.8 (-1 to -0.59) |
| Bahrain | 10 (6 to 16) | 30 (18 to 48) | 2.07 (1.72 to 2.46) | 1.92 (1.12 to 3.14) | 1.96 (1.15 to 3.11) | -0.26 (-0.44 to -0.07) | 2.13 (1.29 to 3.41) | 1.98 (1.19 to 3.11) | -0.6 (-0.78 to -0.42) |
| Bangladesh | 1223 (702 to 1955) | 2133 (1268 to 3381) | 0.74 (0.58 to 0.94) | 1.12 (0.64 to 1.79) | 1.3 (0.77 to 2.05) | -0.21 (-1 to 0.58) | 1.25 (0.74 to 1.95) | 1.31 (0.78 to 2.08) | -0.41 (-1.03 to 0.21) |
| Barbados | 4 (3 to 7) | 6 (4 to 9) | 0.38 (0.23 to 0.54) | 1.65 (1.02 to 2.65) | 1.93 (1.2 to 2.99) | 0.35 (0.24 to 0.47) | 1.6 (0.97 to 2.58) | 1.68 (1.01 to 2.66) | 0.07 (-0.08 to 0.22) |
| Belarus | 629 (382 to 1002) | 607 (376 to 933) | -0.03 (-0.11 to 0.05) | 6.02 (3.66 to 9.59) | 6.51 (4.03 to 10) | 0.16 (-0.18 to 0.51) | 5.73 (3.46 to 9.21) | 5.57 (3.37 to 8.76) | -0.2 (-0.51 to 0.11) |
| Belgium | 422 (257 to 656) | 602 (375 to 919) | 0.43 (0.31 to 0.55) | 4.23 (2.57 to 6.58) | 5.25 (3.27 to 8.01) | 0.86 (0.61 to 1.12) | 3.64 (2.2 to 5.79) | 3.7 (2.25 to 5.83) | 0.24 (-0.11 to 0.58) |
| Belize | 3 (2 to 5) | 9 (5 to 14) | 1.77 (1.56 to 2.03) | 1.7 (0.99 to 2.8) | 2.05 (1.23 to 3.34) | 0.48 (0.34 to 0.62) | 1.87 (1.13 to 2.98) | 2.15 (1.32 to 3.46) | 0.3 (0.13 to 0.47) |
| Benin | 56 (33 to 95) | 146 (84 to 236) | 1.59 (1.42 to 1.77) | 1.16 (0.69 to 1.96) | 1.08 (0.62 to 1.75) | -0.27 (-0.31 to -0.22) | 1.44 (0.87 to 2.28) | 1.41 (0.85 to 2.22) | -0.12 (-0.18 to -0.07) |
| Bermuda | 1 (1 to 2) | 1 (1 to 2) | 0.3 (0.18 to 0.46) | 1.81 (1.09 to 2.87) | 2.2 (1.36 to 3.41) | 0.57 (0.51 to 0.63) | 1.79 (1.08 to 2.87) | 1.78 (1.06 to 2.85) | -0.07 (-0.19 to 0.04) |
| Bhutan | 9 (5 to 14) | 16 (9 to 25) | 0.85 (0.66 to 1.08) | 1.36 (0.78 to 2.21) | 2.09 (1.25 to 3.29) | 1.21 (0.91 to 1.51) | 1.72 (1.03 to 2.69) | 2.29 (1.39 to 3.58) | 0.78 (0.53 to 1.02) |
| Bosnia and Herzegovina | 233 (139 to 380) | 161 (100 to 256) | -0.31 (-0.38 to -0.23) | 5.18 (3.09 to 8.45) | 4.87 (3.03 to 7.75) | -0.49 (-0.73 to -0.26) | 5.18 (3.1 to 8.45) | 4.35 (2.64 to 7.12) | -0.88 (-1.13 to -0.63) |
| Botswana | 16 (10 to 26) | 36 (22 to 57) | 1.26 (1.05 to 1.51) | 1.2 (0.72 to 1.97) | 1.49 (0.91 to 2.37) | 0.62 (0.45 to 0.78) | 1.51 (0.94 to 2.41) | 1.65 (1.01 to 2.55) | 0.16 (-0.01 to 0.34) |
| Brazil | 6025 (3560 to 9464) | 7894 (4750 to 12226) | 0.31 (0.23 to 0.42) | 4.06 (2.4 to 6.37) | 3.58 (2.16 to 5.55) | -0.53 (-0.69 to -0.37) | 4.43 (2.67 to 6.9) | 3.4 (2.04 to 5.28) | -0.94 (-1.11 to -0.77) |
| Brunei Darussalam | 6 (3 to 9) | 9 (5 to 15) | 0.67 (0.51 to 0.87) | 2.12 (1.26 to 3.42) | 2.03 (1.21 to 3.25) | -0.15 (-0.19 to -0.1) | 2.5 (1.51 to 3.92) | 2.11 (1.28 to 3.35) | -0.56 (-0.6 to -0.53) |
| Bulgaria | 590 (358 to 939) | 408 (251 to 633) | -0.31 (-0.36 to -0.24) | 6.8 (4.12 to 10.81) | 6.01 (3.69 to 9.32) | -0.48 (-0.51 to -0.46) | 6.48 (3.87 to 10.44) | 5.28 (3.11 to 8.43) | -0.73 (-0.76 to -0.7) |
| Burkina Faso | 115 (68 to 187) | 280 (163 to 450) | 1.43 (1.26 to 1.64) | 1.21 (0.71 to 1.96) | 1.23 (0.72 to 1.98) | 0.03 (-0.06 to 0.12) | 1.55 (0.94 to 2.42) | 1.59 (0.96 to 2.49) | 0.08 (0.02 to 0.14) |
| Burundi | 65 (38 to 105) | 160 (101 to 256) | 1.47 (1.08 to 2.37) | 1.16 (0.68 to 1.9) | 1.21 (0.77 to 1.93) | -1.82 (-3.05 to -0.57) | 1.48 (0.89 to 2.35) | 1.6 (1.02 to 2.51) | -1.17 (-2.08 to -0.25) |
| Cambodia | 198 (120 to 327) | 375 (230 to 599) | 0.9 (0.62 to 1.14) | 1.92 (1.17 to 3.18) | 2.2 (1.35 to 3.51) | 0.45 (0.34 to 0.57) | 2.37 (1.48 to 3.75) | 2.54 (1.6 to 3.98) | 0.22 (0.14 to 0.3) |
| Cameroon | 111 (66 to 179) | 348 (207 to 571) | 2.15 (1.89 to 2.39) | 1.06 (0.64 to 1.71) | 1.1 (0.65 to 1.8) | 0.11 (0.07 to 0.15) | 1.39 (0.85 to 2.15) | 1.44 (0.89 to 2.29) | 0.1 (0.06 to 0.13) |
| Canada | 622 (382 to 990) | 1110 (691 to 1732) | 0.78 (0.62 to 1.03) | 2.28 (1.4 to 3.63) | 2.96 (1.85 to 4.62) | 1.08 (0.99 to 1.16) | 2.08 (1.27 to 3.33) | 1.95 (1.18 to 3.04) | -0.06 (-0.13 to 0) |
| Central African Republic | 33 (20 to 55) | 79 (45 to 130) | 1.37 (1.06 to 1.92) | 1.21 (0.72 to 2.01) | 1.43 (0.83 to 2.37) | 0.63 (0.32 to 0.94) | 1.56 (0.93 to 2.46) | 1.73 (1.03 to 2.78) | 0.39 (0.19 to 0.6) |
| Chad | 75 (45 to 124) | 195 (113 to 326) | 1.61 (1.38 to 1.84) | 1.24 (0.74 to 2.05) | 1.1 (0.64 to 1.84) | -0.41 (-0.59 to -0.23) | 1.46 (0.88 to 2.34) | 1.45 (0.89 to 2.31) | -0.04 (-0.17 to 0.1) |
| Chile | 277 (166 to 438) | 521 (320 to 811) | 0.88 (0.72 to 1.09) | 2.08 (1.25 to 3.3) | 2.77 (1.7 to 4.32) | 0.97 (0.83 to 1.12) | 2.21 (1.34 to 3.48) | 2.57 (1.55 to 4.07) | 0.57 (0.4 to 0.73) |
| China | 25546 (15320 to 40100) | 42524 (26780 to 65689) | 0.66 (0.56 to 0.78) | 2.17 (1.3 to 3.41) | 2.99 (1.88 to 4.62) | 0.67 (0.3 to 1.04) | 2.43 (1.49 to 3.8) | 2.55 (1.56 to 4) | -0.26 (-0.62 to 0.1) |
| Colombia | 1104 (653 to 1807) | 1249 (766 to 1987) | 0.13 (0.03 to 0.24) | 3.4 (2.01 to 5.56) | 2.55 (1.56 to 4.05) | -1.04 (-1.08 to -0.99) | 3.71 (2.27 to 5.94) | 2.5 (1.52 to 4.05) | -1.37 (-1.41 to -1.32) |
| Comoros | 5 (3 to 8) | 8 (5 to 13) | 0.65 (0.51 to 0.8) | 1.1 (0.67 to 1.78) | 1.13 (0.68 to 1.77) | 0.07 (-0.05 to 0.18) | 1.4 (0.88 to 2.2) | 1.3 (0.81 to 2.03) | -0.25 (-0.34 to -0.16) |
| Congo | 28 (17 to 45) | 60 (37 to 96) | 1.12 (0.92 to 1.45) | 1.17 (0.69 to 1.88) | 1.11 (0.69 to 1.79) | -0.85 (-1.6 to -0.1) | 1.54 (0.95 to 2.39) | 1.39 (0.87 to 2.17) | -0.82 (-1.36 to -0.28) |
| Cook Islands | 0 (0 to 1) | 0 (0 to 1) | 0.12 (-0.01 to 0.27) | 1.7 (1.01 to 2.74) | 2.04 (1.26 to 3.18) | 0.64 (0.19 to 1.08) | 1.88 (1.14 to 2.96) | 1.83 (1.11 to 2.87) | -0.04 (-0.46 to 0.38) |
| Costa Rica | 82 (49 to 135) | 133 (81 to 212) | 0.62 (0.48 to 0.82) | 2.7 (1.61 to 4.45) | 2.8 (1.71 to 4.46) | 0.04 (0 to 0.07) | 2.99 (1.82 to 4.8) | 2.71 (1.64 to 4.36) | -0.36 (-0.41 to -0.31) |
| Croatia | 339 (206 to 539) | 336 (209 to 511) | -0.01 (-0.09 to 0.1) | 6.97 (4.23 to 11.09) | 7.98 (4.97 to 12.14) | 0.46 (0.35 to 0.58) | 6.63 (3.98 to 10.68) | 5.58 (3.35 to 8.83) | -0.59 (-0.74 to -0.45) |
| Cuba | 290 (174 to 464) | 467 (291 to 715) | 0.61 (0.46 to 0.79) | 2.67 (1.61 to 4.28) | 4.14 (2.58 to 6.35) | 1.37 (1.34 to 1.4) | 2.71 (1.64 to 4.32) | 3.06 (1.87 to 4.8) | 0.31 (0.22 to 0.39) |
| Cyprus | 24 (15 to 38) | 45 (28 to 70) | 0.86 (0.71 to 1.03) | 3.1 (1.88 to 4.85) | 3.3 (2.05 to 5.18) | 0.22 (0.04 to 0.39) | 3.16 (1.91 to 4.94) | 2.92 (1.78 to 4.65) | -0.2 (-0.38 to -0.02) |
| Democratic People's Republic of Korea | 332 (200 to 528) | 411 (255 to 638) | 0.24 (0.11 to 0.41) | 1.61 (0.97 to 2.57) | 1.56 (0.97 to 2.42) | -0.01 (-0.09 to 0.06) | 1.74 (1.06 to 2.78) | 1.37 (0.84 to 2.12) | -0.67 (-0.72 to -0.62) |
| Democratic Republic of the Congo | 447 (263 to 745) | 1003 (600 to 1628) | 1.24 (1.05 to 1.46) | 1.17 (0.69 to 1.95) | 1.11 (0.67 to 1.81) | -0.53 (-0.88 to -0.18) | 1.47 (0.9 to 2.31) | 1.46 (0.9 to 2.29) | -0.26 (-0.5 to -0.02) |
| Denmark | 202 (125 to 314) | 186 (114 to 289) | -0.08 (-0.15 to 0) | 3.93 (2.42 to 6.1) | 3.18 (1.95 to 4.94) | -0.89 (-0.98 to -0.81) | 3.16 (1.9 to 5) | 2.51 (1.51 to 4.06) | -0.85 (-0.97 to -0.73) |
| Djibouti | 5 (3 to 8) | 13 (8 to 21) | 1.8 (1.38 to 2.13) | 1.16 (0.67 to 1.94) | 1.07 (0.64 to 1.7) | -0.44 (-0.65 to -0.22) | 1.44 (0.86 to 2.34) | 1.32 (0.8 to 2.07) | -0.43 (-0.57 to -0.29) |
| Dominica | 1 (1 to 2) | 1 (1 to 2) | 0.08 (-0.02 to 0.19) | 1.55 (0.91 to 2.45) | 1.8 (1.11 to 2.78) | 0.64 (0.38 to 0.91) | 1.62 (0.96 to 2.55) | 1.7 (1.04 to 2.67) | 0.37 (0.05 to 0.68) |
| Dominican Republic | 108 (64 to 177) | 218 (131 to 350) | 1.01 (0.84 to 1.21) | 1.52 (0.9 to 2.48) | 1.98 (1.19 to 3.18) | 0.89 (0.71 to 1.07) | 1.69 (1.02 to 2.7) | 2 (1.2 to 3.19) | 0.57 (0.39 to 0.75) |
| Ecuador | 225 (134 to 362) | 463 (281 to 732) | 1.06 (0.9 to 1.23) | 2.26 (1.34 to 3.63) | 2.56 (1.55 to 4.05) | 0.38 (0.22 to 0.54) | 2.55 (1.56 to 4.03) | 2.6 (1.58 to 4.09) | 0.02 (-0.14 to 0.18) |
| Egypt | 1343 (800 to 2210) | 2023 (1216 to 3187) | 0.51 (0.38 to 0.64) | 2.43 (1.45 to 3.99) | 1.91 (1.15 to 3.02) | -0.74 (-0.8 to -0.68) | 2.68 (1.63 to 4.31) | 2.13 (1.31 to 3.34) | -0.67 (-0.72 to -0.61) |
| El Salvador | 185 (113 to 306) | 194 (118 to 301) | 0.04 (-0.09 to 0.18) | 3.5 (2.13 to 5.76) | 3 (1.83 to 4.66) | -0.3 (-0.41 to -0.19) | 3.7 (2.31 to 5.99) | 3.01 (1.84 to 4.64) | -0.55 (-0.63 to -0.47) |
| Equatorial Guinea | 5 (3 to 8) | 14 (8 to 22) | 1.8 (1.54 to 2.06) | 1.19 (0.71 to 1.94) | 0.93 (0.54 to 1.47) | -0.86 (-0.92 to -0.8) | 1.49 (0.9 to 2.35) | 1.28 (0.77 to 1.96) | -0.53 (-0.57 to -0.5) |
| Eritrea | 119 (57 to 230) | 86 (54 to 140) | -0.27 (-0.57 to 0.15) | 3.48 (1.68 to 6.76) | 1.31 (0.82 to 2.12) | -1.92 (-2.57 to -1.27) | 3.54 (1.83 to 6.43) | 1.8 (1.15 to 2.88) | -1.29 (-1.74 to -0.83) |
| Estonia | 120 (74 to 189) | 67 (41 to 105) | -0.44 (-0.48 to -0.39) | 7.67 (4.7 to 12.05) | 5.12 (3.12 to 8.02) | -1.67 (-1.81 to -1.53) | 7.12 (4.33 to 11.35) | 4.27 (2.53 to 6.82) | -1.99 (-2.1 to -1.87) |
| Ethiopia | 1130 (604 to 1963) | 1195 (691 to 1956) | 0.06 (-0.24 to 0.3) | 2.24 (1.19 to 3.88) | 1.1 (0.63 to 1.8) | -1.74 (-2.14 to -1.33) | 2.58 (1.47 to 4.22) | 1.41 (0.87 to 2.25) | -1.6 (-1.86 to -1.34) |
| Fiji | 10 (6 to 15) | 12 (7 to 18) | 0.24 (0.12 to 0.38) | 1.26 (0.74 to 1.99) | 1.27 (0.75 to 2) | -0.05 (-0.19 to 0.09) | 1.45 (0.88 to 2.28) | 1.36 (0.8 to 2.12) | -0.32 (-0.43 to -0.2) |
| Finland | 243 (148 to 381) | 294 (181 to 453) | 0.21 (0.12 to 0.33) | 4.85 (2.95 to 7.6) | 5.32 (3.26 to 8.18) | 0.16 (-0.29 to 0.61) | 4.31 (2.59 to 6.89) | 3.83 (2.27 to 6.14) | -0.49 (-0.98 to 0.01) |
| France | 2520 (1509 to 3917) | 3128 (1946 to 4767) | 0.24 (0.16 to 0.36) | 4.36 (2.61 to 6.78) | 4.71 (2.93 to 7.18) | 0.33 (0.25 to 0.4) | 3.77 (2.26 to 5.92) | 3.33 (1.98 to 5.28) | -0.32 (-0.43 to -0.21) |
| Gabon | 13 (8 to 21) | 21 (13 to 34) | 0.65 (0.51 to 0.78) | 1.32 (0.81 to 2.11) | 1.18 (0.71 to 1.89) | -0.41 (-0.44 to -0.37) | 1.62 (1.01 to 2.54) | 1.45 (0.89 to 2.27) | -0.41 (-0.43 to -0.39) |
| Georgia | 242 (144 to 382) | 178 (107 to 282) | -0.26 (-0.32 to -0.19) | 4.38 (2.62 to 6.92) | 4.95 (2.98 to 7.81) | 0.65 (0.46 to 0.84) | 4.29 (2.56 to 6.85) | 4.52 (2.69 to 7.28) | 0.38 (0.18 to 0.57) |
| Germany | 2861 (1750 to 4493) | 3284 (2022 to 5014) | 0.15 (0.05 to 0.27) | 3.58 (2.19 to 5.62) | 3.85 (2.37 to 5.87) | 0.24 (0.18 to 0.3) | 3.03 (1.82 to 4.9) | 2.72 (1.61 to 4.27) | -0.38 (-0.44 to -0.32) |
| Ghana | 152 (89 to 249) | 368 (221 to 594) | 1.42 (1.25 to 1.62) | 1.02 (0.59 to 1.67) | 1.07 (0.65 to 1.73) | 0.12 (0.04 to 0.21) | 1.33 (0.81 to 2.11) | 1.38 (0.84 to 2.16) | 0.05 (-0.01 to 0.12) |
| Greece | 370 (226 to 576) | 297 (184 to 457) | -0.2 (-0.27 to -0.1) | 3.56 (2.18 to 5.55) | 2.92 (1.81 to 4.49) | -0.64 (-0.72 to -0.56) | 3.22 (1.94 to 5.1) | 2.37 (1.43 to 3.81) | -0.97 (-1.08 to -0.85) |
| Greenland | 2 (1 to 2) | 2 (1 to 3) | 0.14 (0.05 to 0.27) | 2.79 (1.66 to 4.47) | 3.16 (1.9 to 4.91) | 0.35 (0.24 to 0.46) | 3.49 (2.13 to 5.46) | 2.84 (1.72 to 4.45) | -0.79 (-0.88 to -0.7) |
| Grenada | 2 (1 to 3) | 2 (1 to 3) | 0.35 (0.24 to 0.49) | 1.84 (1.11 to 2.99) | 2.11 (1.28 to 3.35) | 0.45 (0.34 to 0.55) | 1.95 (1.18 to 3.12) | 2.06 (1.25 to 3.29) | 0.19 (0.07 to 0.32) |
| Guam | 2 (1 to 3) | 3 (2 to 4) | 0.37 (0.23 to 0.59) | 1.5 (0.9 to 2.42) | 1.76 (1.08 to 2.79) | 0.52 (0.44 to 0.6) | 1.68 (1.04 to 2.69) | 1.61 (0.98 to 2.55) | -0.13 (-0.24 to -0.03) |
| Guatemala | 261 (154 to 434) | 486 (289 to 792) | 0.86 (0.67 to 1.06) | 3.11 (1.84 to 5.18) | 3.08 (1.83 to 5.02) | 0.01 (-0.03 to 0.05) | 3.55 (2.17 to 5.66) | 3.32 (2 to 5.32) | -0.22 (-0.26 to -0.17) |
| Guinea | 73 (44 to 119) | 149 (88 to 239) | 1.05 (0.9 to 1.21) | 1.22 (0.74 to 1.99) | 1.11 (0.66 to 1.78) | -0.29 (-0.4 to -0.19) | 1.4 (0.86 to 2.27) | 1.41 (0.86 to 2.17) | 0.02 (-0.05 to 0.1) |
| Guinea-Bissau | 13 (8 to 22) | 23 (14 to 38) | 0.73 (0.6 to 0.87) | 1.33 (0.79 to 2.17) | 1.12 (0.65 to 1.83) | -0.72 (-0.95 to -0.49) | 1.72 (1.07 to 2.7) | 1.56 (0.95 to 2.45) | -0.43 (-0.58 to -0.27) |
| Guyana | 15 (9 to 24) | 18 (11 to 28) | 0.24 (0.15 to 0.34) | 1.87 (1.13 to 3.03) | 2.36 (1.41 to 3.68) | 0.63 (0.51 to 0.75) | 2.2 (1.35 to 3.5) | 2.47 (1.5 to 3.85) | 0.2 (0.07 to 0.33) |
| Haiti | 124 (75 to 200) | 301 (185 to 474) | 1.43 (1.1 to 2.01) | 1.94 (1.17 to 3.13) | 2.34 (1.44 to 3.68) | 0.67 (-0.53 to 1.89) | 2.21 (1.37 to 3.52) | 2.54 (1.61 to 3.88) | 0.59 (-0.52 to 1.71) |
| Honduras | 123 (72 to 204) | 232 (141 to 377) | 0.89 (0.74 to 1.11) | 2.61 (1.54 to 4.32) | 2.3 (1.39 to 3.73) | -0.71 (-1.19 to -0.22) | 2.83 (1.7 to 4.52) | 2.53 (1.56 to 4.01) | -0.61 (-1.01 to -0.21) |
| Hungary | 917 (568 to 1428) | 646 (403 to 1018) | -0.3 (-0.34 to -0.24) | 8.82 (5.46 to 13.73) | 6.73 (4.2 to 10.61) | -1.22 (-1.35 to -1.09) | 7.65 (4.67 to 12.02) | 5.11 (3.08 to 8.25) | -1.59 (-1.71 to -1.47) |
| Iceland | 8 (5 to 12) | 11 (6 to 17) | 0.38 (0.25 to 0.53) | 3.11 (1.91 to 4.91) | 3.1 (1.85 to 4.87) | -0.01 (-0.07 to 0.06) | 2.99 (1.81 to 4.7) | 2.67 (1.57 to 4.35) | -0.35 (-0.44 to -0.25) |
| India | 21600 (12697 to 34390) | 38458 (23352 to 58666) | 0.78 (0.68 to 0.9) | 2.53 (1.49 to 4.03) | 2.72 (1.65 to 4.15) | 0.09 (0.04 to 0.14) | 3.28 (2.01 to 5.15) | 3.02 (1.86 to 4.58) | -0.4 (-0.44 to -0.35) |
| Indonesia | 3499 (2103 to 5615) | 4364 (2677 to 6863) | 0.25 (0.18 to 0.32) | 1.89 (1.14 to 3.04) | 1.56 (0.96 to 2.46) | -0.78 (-0.89 to -0.66) | 2.28 (1.4 to 3.57) | 1.71 (1.06 to 2.69) | -1.08 (-1.18 to -0.97) |
| Iraq | 652 (416 to 1050) | 1164 (745 to 1873) | 0.79 (0.65 to 0.96) | 3.54 (2.26 to 5.7) | 2.82 (1.81 to 4.54) | -0.31 (-0.67 to 0.05) | 3.97 (2.56 to 6.32) | 3.04 (1.98 to 4.82) | -0.52 (-0.83 to -0.2) |
| Ireland | 101 (59 to 159) | 149 (91 to 234) | 0.48 (0.35 to 0.62) | 2.81 (1.63 to 4.43) | 3.02 (1.85 to 4.73) | 0.15 (-0.02 to 0.32) | 2.74 (1.59 to 4.34) | 2.67 (1.59 to 4.33) | -0.1 (-0.3 to 0.11) |
| Israel | 113 (67 to 180) | 223 (136 to 356) | 0.97 (0.81 to 1.18) | 2.28 (1.35 to 3.63) | 2.33 (1.41 to 3.71) | 0.1 (-0.11 to 0.31) | 2.3 (1.37 to 3.64) | 2.21 (1.33 to 3.56) | -0.08 (-0.29 to 0.14) |
| Italy | 2533 (1562 to 3987) | 2392 (1519 to 3658) | -0.06 (-0.1 to 0) | 4.46 (2.75 to 7.02) | 4 (2.54 to 6.12) | -0.5 (-0.54 to -0.45) | 3.79 (2.28 to 6.01) | 2.95 (1.75 to 4.72) | -0.88 (-0.92 to -0.85) |
| Jamaica | 42 (25 to 68) | 54 (32 to 86) | 0.29 (0.17 to 0.42) | 1.76 (1.05 to 2.86) | 1.92 (1.16 to 3.08) | 0.17 (0.05 to 0.29) | 1.84 (1.1 to 2.96) | 1.84 (1.1 to 2.98) | -0.11 (-0.24 to 0.02) |
| Japan | 3064 (1872 to 4836) | 3034 (1898 to 4634) | -0.01 (-0.07 to 0.07) | 2.43 (1.49 to 3.84) | 2.38 (1.49 to 3.63) | -0.21 (-0.38 to -0.04) | 2.21 (1.33 to 3.55) | 1.62 (0.98 to 2.61) | -1.11 (-1.29 to -0.93) |
| Jordan | 77 (44 to 123) | 224 (129 to 358) | 1.92 (1.68 to 2.2) | 2.06 (1.19 to 3.29) | 1.82 (1.05 to 2.91) | -0.45 (-0.51 to -0.38) | 2.36 (1.42 to 3.74) | 1.9 (1.12 to 2.99) | -0.71 (-0.77 to -0.64) |
| Kazakhstan | 671 (398 to 1061) | 689 (418 to 1064) | 0.03 (-0.05 to 0.11) | 4.09 (2.43 to 6.47) | 3.64 (2.21 to 5.61) | -0.29 (-0.43 to -0.16) | 4.21 (2.51 to 6.63) | 3.66 (2.22 to 5.67) | -0.37 (-0.48 to -0.26) |
| Kenya | 238 (141 to 385) | 506 (301 to 803) | 1.12 (1.05 to 1.21) | 1.03 (0.61 to 1.66) | 1.01 (0.6 to 1.6) | -0.25 (-0.4 to -0.09) | 1.42 (0.87 to 2.21) | 1.38 (0.84 to 2.13) | -0.22 (-0.37 to -0.07) |
| Kiribati | 1 (1 to 1) | 1 (1 to 2) | 0.47 (0.33 to 0.63) | 1.11 (0.67 to 1.74) | 1 (0.61 to 1.57) | -0.3 (-0.45 to -0.15) | 1.33 (0.82 to 2.07) | 1.14 (0.71 to 1.78) | -0.46 (-0.59 to -0.33) |
| Kuwait | 55 (30 to 93) | 116 (71 to 185) | 1.11 (0.63 to 1.52) | 3.2 (1.74 to 5.4) | 2.5 (1.53 to 3.97) | -0.54 (-0.77 to -0.32) | 3.32 (1.83 to 5.46) | 2.42 (1.48 to 3.8) | -0.78 (-0.99 to -0.57) |
| Kyrgyzstan | 161 (96 to 258) | 162 (96 to 259) | 0 (-0.07 to 0.09) | 3.61 (2.15 to 5.78) | 2.35 (1.4 to 3.78) | -1.56 (-1.62 to -1.49) | 3.81 (2.28 to 6.02) | 2.46 (1.48 to 3.94) | -1.57 (-1.63 to -1.5) |
| Lao People's Democratic Republic | 69 (42 to 113) | 103 (62 to 161) | 0.49 (0.28 to 0.67) | 1.66 (1 to 2.71) | 1.4 (0.84 to 2.18) | -0.34 (-0.43 to -0.25) | 1.99 (1.22 to 3.22) | 1.59 (0.97 to 2.43) | -0.62 (-0.67 to -0.57) |
| Latvia | 234 (142 to 366) | 107 (68 to 165) | -0.54 (-0.57 to -0.5) | 8.81 (5.36 to 13.76) | 5.74 (3.62 to 8.81) | -1.94 (-2.15 to -1.72) | 8.01 (4.84 to 12.69) | 4.6 (2.79 to 7.37) | -2.33 (-2.53 to -2.13) |
| Lebanon | 94 (58 to 155) | 125 (78 to 197) | 0.32 (0.1 to 0.56) | 3.15 (1.94 to 5.19) | 2.25 (1.41 to 3.55) | -0.95 (-1.19 to -0.7) | 3.47 (2.16 to 5.62) | 2.15 (1.35 to 3.43) | -1.47 (-1.74 to -1.19) |
| Lesotho | 19 (11 to 31) | 30 (18 to 49) | 0.6 (0.46 to 0.78) | 1.24 (0.73 to 2.02) | 1.62 (0.96 to 2.6) | 0.94 (0.8 to 1.09) | 1.49 (0.91 to 2.34) | 1.88 (1.14 to 2.92) | 0.87 (0.72 to 1.03) |
| Liberia | 54 (27 to 102) | 55 (34 to 90) | 0.03 (-0.4 to 0.49) | 2.19 (1.09 to 4.14) | 1.01 (0.63 to 1.65) | -2.12 (-2.77 to -1.47) | 2.22 (1.17 to 3.93) | 1.32 (0.84 to 2.08) | -1.3 (-1.8 to -0.79) |
| Libya | 100 (59 to 162) | 194 (118 to 303) | 0.94 (0.77 to 1.2) | 2.37 (1.41 to 3.84) | 2.82 (1.72 to 4.41) | 1.11 (0.79 to 1.44) | 2.74 (1.66 to 4.36) | 2.98 (1.78 to 4.64) | 0.88 (0.56 to 1.21) |
| Lithuania | 279 (172 to 446) | 178 (110 to 273) | -0.36 (-0.41 to -0.3) | 7.58 (4.69 to 12.14) | 6.51 (4.04 to 9.99) | -0.69 (-0.87 to -0.51) | 7.07 (4.34 to 11.38) | 4.97 (2.99 to 7.84) | -1.36 (-1.54 to -1.18) |
| Luxembourg | 16 (10 to 24) | 24 (15 to 38) | 0.54 (0.41 to 0.69) | 4.14 (2.54 to 6.42) | 3.76 (2.35 to 5.93) | -0.27 (-0.32 to -0.21) | 3.65 (2.21 to 5.68) | 3.11 (1.89 to 4.94) | -0.51 (-0.56 to -0.45) |
| Madagascar | 128 (75 to 206) | 248 (144 to 396) | 0.94 (0.8 to 1.09) | 1.07 (0.63 to 1.73) | 0.87 (0.5 to 1.39) | -0.71 (-0.77 to -0.65) | 1.28 (0.77 to 1.99) | 1.1 (0.66 to 1.73) | -0.54 (-0.6 to -0.49) |
| Malawi | 102 (60 to 165) | 180 (106 to 283) | 0.77 (0.65 to 0.91) | 1.04 (0.61 to 1.69) | 0.92 (0.54 to 1.46) | -0.48 (-0.54 to -0.42) | 1.28 (0.78 to 1.97) | 1.22 (0.72 to 1.87) | -0.23 (-0.3 to -0.17) |
| Malaysia | 249 (149 to 402) | 520 (310 to 816) | 1.08 (0.93 to 1.31) | 1.41 (0.84 to 2.28) | 1.63 (0.97 to 2.57) | 0.43 (0.39 to 0.48) | 1.71 (1.03 to 2.74) | 1.68 (1.01 to 2.63) | -0.1 (-0.12 to -0.07) |
| Maldives | 3 (2 to 6) | 9 (5 to 14) | 1.52 (1.24 to 1.85) | 1.52 (0.9 to 2.5) | 1.65 (0.97 to 2.62) | 0.36 (-0.03 to 0.75) | 1.91 (1.13 to 3.03) | 1.75 (1.06 to 2.77) | -0.24 (-0.57 to 0.09) |
| Mali | 115 (67 to 191) | 294 (172 to 484) | 1.56 (1.37 to 1.74) | 1.33 (0.77 to 2.21) | 1.22 (0.72 to 2.01) | -0.45 (-0.84 to -0.07) | 1.63 (1 to 2.6) | 1.61 (0.96 to 2.54) | -0.19 (-0.46 to 0.09) |
| Malta | 12 (7 to 18) | 16 (10 to 24) | 0.35 (0.22 to 0.5) | 3.15 (1.88 to 4.92) | 3.57 (2.19 to 5.53) | 0.52 (0.34 to 0.69) | 3.07 (1.83 to 4.77) | 2.85 (1.69 to 4.55) | -0.07 (-0.3 to 0.17) |
| Marshall Islands | 1 (0 to 1) | 1 (1 to 1) | 0.44 (0.31 to 0.6) | 1.3 (0.75 to 2.1) | 1.51 (0.92 to 2.43) | 0.48 (0.41 to 0.55) | 1.76 (1.07 to 2.74) | 1.71 (1.07 to 2.74) | -0.09 (-0.17 to -0.02) |
| Mauritania | 26 (15 to 42) | 48 (29 to 77) | 0.86 (0.71 to 1) | 1.26 (0.75 to 2.05) | 1.09 (0.66 to 1.76) | -0.53 (-0.58 to -0.48) | 1.6 (0.99 to 2.55) | 1.38 (0.85 to 2.18) | -0.56 (-0.6 to -0.52) |
| Mauritius | 15 (9 to 23) | 20 (12 to 33) | 0.39 (0.24 to 0.56) | 1.34 (0.82 to 2.14) | 1.6 (0.98 to 2.58) | 0.85 (0.75 to 0.94) | 1.43 (0.89 to 2.26) | 1.42 (0.85 to 2.26) | 0.22 (0.12 to 0.31) |
| Mexico | 3604 (2110 to 5850) | 3908 (2357 to 6138) | 0.08 (0.02 to 0.18) | 4.22 (2.47 to 6.85) | 3.02 (1.82 to 4.75) | 0.04 (-0.42 to 0.5) | 4.78 (2.88 to 7.62) | 3.03 (1.82 to 4.76) | -0.46 (-0.89 to -0.03) |
| Mongolia | 67 (39 to 109) | 125 (75 to 200) | 0.87 (0.73 to 1.02) | 3.1 (1.81 to 5.06) | 3.75 (2.26 to 5.98) | 0.81 (0.69 to 0.93) | 3.52 (2.1 to 5.58) | 3.92 (2.38 to 6.23) | 0.48 (0.41 to 0.56) |
| Montenegro | 36 (21 to 58) | 33 (20 to 52) | -0.08 (-0.14 to -0.01) | 5.73 (3.42 to 9.24) | 5.32 (3.23 to 8.48) | -0.21 (-0.3 to -0.12) | 5.7 (3.4 to 9.2) | 4.84 (2.9 to 7.77) | -0.51 (-0.59 to -0.44) |
| Morocco | 703 (417 to 1152) | 975 (578 to 1564) | 0.39 (0.27 to 0.51) | 2.77 (1.64 to 4.54) | 2.62 (1.55 to 4.21) | -0.32 (-0.4 to -0.23) | 3.08 (1.85 to 5.02) | 2.67 (1.58 to 4.27) | -0.57 (-0.66 to -0.48) |
| Mozambique | 190 (114 to 313) | 345 (209 to 561) | 0.82 (0.49 to 1.1) | 1.42 (0.85 to 2.34) | 1.11 (0.67 to 1.81) | -0.69 (-0.81 to -0.57) | 1.66 (1.03 to 2.66) | 1.52 (0.96 to 2.36) | -0.16 (-0.25 to -0.07) |
| Myanmar | 831 (487 to 1362) | 1240 (776 to 1975) | 0.49 (0.37 to 0.65) | 2.06 (1.2 to 3.37) | 2.2 (1.38 to 3.5) | 0.08 (-0.66 to 0.82) | 2.34 (1.39 to 3.72) | 2.31 (1.45 to 3.66) | -0.13 (-0.82 to 0.56) |
| Namibia | 18 (11 to 29) | 32 (19 to 51) | 0.8 (0.61 to 1.02) | 1.27 (0.77 to 2.05) | 1.32 (0.8 to 2.11) | 0.02 (-0.08 to 0.13) | 1.59 (0.99 to 2.47) | 1.53 (0.93 to 2.38) | -0.27 (-0.39 to -0.15) |
| Nepal | 442 (258 to 696) | 897 (541 to 1389) | 1.03 (0.87 to 1.21) | 2.27 (1.33 to 3.58) | 2.88 (1.74 to 4.46) | 0.74 (0.47 to 1.02) | 2.78 (1.68 to 4.29) | 3.14 (1.93 to 4.83) | 0.34 (0.11 to 0.56) |
| Netherlands | 358 (222 to 563) | 612 (383 to 950) | 0.71 (0.54 to 0.9) | 2.4 (1.49 to 3.77) | 3.56 (2.22 to 5.52) | 1.68 (1.1 to 2.27) | 2.16 (1.32 to 3.49) | 2.42 (1.46 to 3.88) | 0.67 (0.22 to 1.13) |
| New Zealand | 145 (89 to 229) | 211 (128 to 328) | 0.46 (0.36 to 0.57) | 4.24 (2.59 to 6.71) | 4.09 (2.48 to 6.35) | -0.06 (-0.21 to 0.08) | 4.11 (2.5 to 6.52) | 3.68 (2.21 to 5.84) | -0.33 (-0.52 to -0.13) |
| Nicaragua | 102 (63 to 167) | 148 (91 to 238) | 0.45 (0.29 to 0.62) | 2.63 (1.61 to 4.3) | 2.22 (1.37 to 3.57) | -0.63 (-0.88 to -0.39) | 2.82 (1.76 to 4.46) | 2.43 (1.53 to 3.85) | -0.54 (-0.74 to -0.34) |
| Niger | 97 (58 to 160) | 283 (164 to 470) | 1.93 (1.71 to 2.13) | 1.2 (0.72 to 1.99) | 1.13 (0.66 to 1.88) | -0.19 (-0.24 to -0.13) | 1.55 (0.95 to 2.42) | 1.53 (0.93 to 2.4) | -0.01 (-0.06 to 0.03) |
| Nigeria | 1086 (648 to 1759) | 2398 (1416 to 3859) | 1.21 (1.13 to 1.29) | 1.21 (0.72 to 1.95) | 1.04 (0.61 to 1.67) | -0.54 (-0.62 to -0.47) | 1.47 (0.9 to 2.3) | 1.38 (0.84 to 2.13) | -0.25 (-0.32 to -0.18) |
| Northern Mariana Islands | 1 (1 to 1) | 1 (1 to 2) | 0.21 (0.09 to 0.38) | 2.09 (1.22 to 3.29) | 2.34 (1.39 to 3.68) | 0.32 (0.26 to 0.38) | 2.4 (1.45 to 3.76) | 2.36 (1.41 to 3.72) | -0.1 (-0.16 to -0.04) |
| Norway | 167 (103 to 263) | 192 (120 to 302) | 0.15 (0.1 to 0.21) | 3.93 (2.43 to 6.18) | 3.54 (2.22 to 5.57) | -0.38 (-0.53 to -0.23) | 3.17 (1.93 to 5.02) | 2.6 (1.58 to 4.09) | -0.62 (-0.8 to -0.44) |
| Oman | 65 (39 to 106) | 130 (78 to 210) | 0.99 (0.81 to 1.2) | 3.3 (1.96 to 5.35) | 2.77 (1.66 to 4.46) | -0.57 (-0.72 to -0.41) | 4.17 (2.53 to 6.46) | 3.2 (1.94 to 5.06) | -1 (-1.14 to -0.86) |
| Pakistan | 1351 (794 to 2176) | 2617 (1580 to 4170) | 0.94 (0.81 to 1.09) | 1.22 (0.71 to 1.96) | 1.11 (0.67 to 1.77) | -0.21 (-0.52 to 0.11) | 1.43 (0.87 to 2.25) | 1.28 (0.79 to 2) | -0.31 (-0.59 to -0.03) |
| Palestine | 51 (32 to 84) | 118 (73 to 189) | 1.3 (1.04 to 1.57) | 2.5 (1.54 to 4.12) | 2.29 (1.42 to 3.69) | -0.1 (-0.7 to 0.49) | 2.79 (1.76 to 4.44) | 2.56 (1.61 to 4.02) | -0.12 (-0.57 to 0.33) |
| Panama | 62 (36 to 103) | 99 (60 to 161) | 0.6 (0.47 to 0.76) | 2.59 (1.51 to 4.29) | 2.3 (1.4 to 3.75) | -0.45 (-0.51 to -0.39) | 2.74 (1.61 to 4.46) | 2.29 (1.38 to 3.74) | -0.65 (-0.71 to -0.59) |
| Papua New Guinea | 62 (37 to 98) | 201 (120 to 315) | 2.24 (1.99 to 2.56) | 1.51 (0.91 to 2.4) | 1.92 (1.15 to 3.01) | 0.42 (-0.03 to 0.86) | 2.01 (1.26 to 3.08) | 2.49 (1.53 to 3.86) | 0.44 (0.13 to 0.74) |
| Paraguay | 113 (67 to 184) | 194 (114 to 309) | 0.71 (0.57 to 0.9) | 2.8 (1.65 to 4.56) | 2.71 (1.59 to 4.32) | -0.13 (-0.23 to -0.03) | 3.04 (1.82 to 4.85) | 2.77 (1.64 to 4.42) | -0.31 (-0.41 to -0.22) |
| Peru | 458 (269 to 765) | 760 (454 to 1218) | 0.66 (0.45 to 0.87) | 2.11 (1.24 to 3.53) | 2.1 (1.25 to 3.36) | 0.13 (0.06 to 0.21) | 2.28 (1.37 to 3.68) | 2.1 (1.26 to 3.36) | -0.18 (-0.23 to -0.13) |
| Philippines | 1147 (677 to 1845) | 1658 (999 to 2615) | 0.45 (0.35 to 0.55) | 1.82 (1.08 to 2.93) | 1.46 (0.88 to 2.31) | -0.51 (-0.7 to -0.33) | 2.14 (1.3 to 3.37) | 1.6 (0.98 to 2.5) | -0.79 (-0.93 to -0.65) |
| Poland | 2461 (1483 to 3938) | 2242 (1381 to 3514) | -0.09 (-0.13 to -0.04) | 6.45 (3.88 to 10.32) | 5.86 (3.61 to 9.19) | -0.38 (-0.48 to -0.29) | 6.22 (3.75 to 9.97) | 4.74 (2.83 to 7.63) | -0.97 (-1.07 to -0.87) |
| Portugal | 328 (201 to 520) | 287 (180 to 440) | -0.13 (-0.21 to -0.01) | 3.24 (1.98 to 5.13) | 2.7 (1.69 to 4.14) | -0.77 (-0.86 to -0.68) | 2.99 (1.82 to 4.8) | 1.94 (1.17 to 3.02) | -1.59 (-1.66 to -1.51) |
| Puerto Rico | 85 (51 to 139) | 110 (68 to 171) | 0.29 (0.17 to 0.46) | 2.35 (1.41 to 3.84) | 3.33 (2.06 to 5.2) | 1.15 (1.08 to 1.22) | 2.36 (1.42 to 3.86) | 2.51 (1.52 to 4.04) | 0.27 (0.14 to 0.41) |
| Qatar | 14 (8 to 22) | 85 (50 to 137) | 5.27 (4.55 to 6.12) | 3.04 (1.77 to 4.87) | 2.85 (1.67 to 4.6) | 0.02 (-0.14 to 0.18) | 3.19 (1.91 to 5.01) | 2.82 (1.65 to 4.45) | -0.37 (-0.5 to -0.25) |
| Republic of Korea | 1481 (894 to 2346) | 1675 (1055 to 2624) | 0.13 (0.01 to 0.28) | 3.35 (2.02 to 5.3) | 3.25 (2.05 to 5.09) | -0.35 (-0.42 to -0.29) | 3.56 (2.19 to 5.58) | 2.5 (1.54 to 4) | -1.39 (-1.48 to -1.3) |
| Republic of Moldova | 263 (159 to 421) | 150 (93 to 236) | -0.43 (-0.48 to -0.37) | 5.91 (3.58 to 9.47) | 4.17 (2.58 to 6.57) | -1.26 (-1.32 to -1.19) | 5.91 (3.58 to 9.48) | 3.65 (2.19 to 5.78) | -1.71 (-1.77 to -1.65) |
| Romania | 1672 (1007 to 2677) | 1120 (695 to 1741) | -0.33 (-0.38 to -0.26) | 7.15 (4.31 to 11.45) | 5.92 (3.67 to 9.19) | -0.77 (-0.84 to -0.71) | 6.92 (4.13 to 11.14) | 5.06 (3.05 to 8.12) | -1.18 (-1.24 to -1.11) |
| Russian Federation | 10331 (6229 to 16580) | 8807 (5345 to 13737) | -0.15 (-0.18 to -0.11) | 6.84 (4.13 to 10.98) | 6.08 (3.69 to 9.48) | -0.68 (-1.1 to -0.27) | 6.53 (3.93 to 10.54) | 5.27 (3.14 to 8.34) | -0.98 (-1.35 to -0.62) |
| Rwanda | 112 (62 to 191) | 169 (105 to 272) | 0.51 (0.1 to 1.09) | 1.55 (0.86 to 2.65) | 1.27 (0.79 to 2.05) | -1.73 (-2.82 to -0.63) | 1.85 (1.06 to 2.98) | 1.66 (1.04 to 2.62) | -1.33 (-2.26 to -0.39) |
| Saint Kitts and Nevis | 1 (0 to 1) | 1 (1 to 2) | 0.61 (0.47 to 0.76) | 1.98 (1.19 to 3.19) | 2.25 (1.38 to 3.61) | 0.5 (0.37 to 0.63) | 2.09 (1.26 to 3.32) | 2.19 (1.33 to 3.54) | 0.24 (0.11 to 0.37) |
| Saint Lucia | 2 (1 to 4) | 4 (2 to 6) | 0.59 (0.44 to 0.79) | 1.63 (0.96 to 2.62) | 1.99 (1.21 to 3.14) | 0.66 (0.55 to 0.76) | 1.8 (1.08 to 2.82) | 1.84 (1.09 to 2.91) | 0.06 (-0.06 to 0.18) |
| Saint Vincent and the Grenadines | 2 (1 to 3) | 2 (1 to 4) | 0.32 (0.2 to 0.46) | 1.65 (0.97 to 2.65) | 2.09 (1.27 to 3.31) | 0.72 (0.6 to 0.85) | 1.79 (1.08 to 2.83) | 1.97 (1.19 to 3.17) | 0.27 (0.13 to 0.4) |
| Samoa | 2 (1 to 4) | 3 (2 to 5) | 0.3 (0.14 to 0.45) | 1.47 (0.86 to 2.42) | 1.51 (0.93 to 2.35) | 0.39 (-0.26 to 1.04) | 1.77 (1.08 to 2.79) | 1.76 (1.1 to 2.72) | 0.19 (-0.33 to 0.72) |
| Sao Tome and Principe | 2 (1 to 3) | 3 (2 to 6) | 1.01 (0.86 to 1.2) | 1.42 (0.83 to 2.34) | 1.6 (0.94 to 2.57) | 0.2 (0.09 to 0.32) | 1.61 (0.96 to 2.55) | 1.94 (1.17 to 3.04) | 0.47 (0.42 to 0.53) |
| Saudi Arabia | 720 (424 to 1164) | 2337 (1389 to 3672) | 2.25 (2 to 2.57) | 4.54 (2.68 to 7.34) | 6.2 (3.68 to 9.74) | 1.14 (1.04 to 1.24) | 5.58 (3.36 to 9.03) | 6.15 (3.74 to 9.57) | 0.4 (0.35 to 0.44) |
| Senegal | 80 (47 to 133) | 162 (98 to 261) | 1.02 (0.89 to 1.2) | 1.05 (0.61 to 1.74) | 1.02 (0.62 to 1.64) | -0.15 (-0.22 to -0.09) | 1.29 (0.8 to 2.02) | 1.3 (0.8 to 2) | -0.04 (-0.09 to 0.02) |
| Serbia | 490 (298 to 778) | 451 (274 to 709) | -0.08 (-0.14 to 0) | 5.09 (3.09 to 8.08) | 5.05 (3.07 to 7.95) | -0.04 (-0.17 to 0.09) | 4.99 (2.98 to 7.93) | 4.35 (2.59 to 7.05) | -0.49 (-0.63 to -0.35) |
| Seychelles | 1 (1 to 2) | 2 (1 to 3) | 0.45 (0.32 to 0.64) | 1.59 (0.94 to 2.56) | 1.59 (0.95 to 2.49) | -0.04 (-0.11 to 0.03) | 1.71 (1.02 to 2.74) | 1.52 (0.9 to 2.37) | -0.43 (-0.5 to -0.36) |
| Sierra Leone | 48 (28 to 79) | 95 (57 to 155) | 0.99 (0.83 to 1.22) | 1.15 (0.67 to 1.89) | 1.07 (0.65 to 1.75) | -1.32 (-1.89 to -0.74) | 1.32 (0.79 to 2.07) | 1.35 (0.84 to 2.15) | -0.77 (-1.21 to -0.32) |
| Singapore | 63 (38 to 102) | 118 (71 to 187) | 0.87 (0.66 to 1.12) | 2.08 (1.26 to 3.35) | 2.07 (1.23 to 3.26) | -0.18 (-0.32 to -0.03) | 2.09 (1.29 to 3.34) | 1.89 (1.12 to 3.02) | -0.48 (-0.64 to -0.31) |
| Slovakia | 391 (240 to 630) | 380 (235 to 602) | -0.03 (-0.09 to 0.05) | 7.4 (4.53 to 11.92) | 7 (4.33 to 11.08) | -0.15 (-0.21 to -0.09) | 7.12 (4.36 to 11.54) | 5.84 (3.51 to 9.36) | -0.6 (-0.66 to -0.55) |
| Slovenia | 183 (113 to 286) | 188 (117 to 289) | 0.03 (-0.04 to 0.12) | 9.27 (5.73 to 14.51) | 9.1 (5.67 to 13.95) | 0.31 (0.11 to 0.51) | 8.51 (5.21 to 13.48) | 6.57 (3.97 to 10.47) | -0.47 (-0.71 to -0.22) |
| Solomon Islands | 6 (4 to 10) | 17 (10 to 27) | 1.68 (1.48 to 1.9) | 1.9 (1.12 to 3.02) | 2.52 (1.5 to 3.95) | 0.94 (0.79 to 1.08) | 2.8 (1.7 to 4.34) | 3.36 (2.08 to 5.19) | 0.6 (0.52 to 0.68) |
| Somalia | 113 (64 to 195) | 253 (152 to 422) | 1.23 (0.79 to 1.59) | 1.43 (0.81 to 2.45) | 1.17 (0.7 to 1.95) | -0.2 (-0.62 to 0.21) | 1.74 (1.03 to 2.86) | 1.58 (0.99 to 2.44) | -0.05 (-0.32 to 0.23) |
| South Africa | 826 (501 to 1318) | 931 (563 to 1463) | 0.13 (0.07 to 0.19) | 2.23 (1.35 to 3.56) | 1.64 (0.99 to 2.57) | -1.2 (-1.34 to -1.05) | 2.61 (1.62 to 4.02) | 1.66 (1.02 to 2.61) | -1.64 (-1.79 to -1.49) |
| South Sudan | 65 (39 to 105) | 109 (67 to 176) | 0.68 (0.51 to 0.89) | 1.1 (0.66 to 1.79) | 1.13 (0.69 to 1.82) | 0.07 (-0.46 to 0.6) | 1.33 (0.8 to 2.1) | 1.38 (0.86 to 2.13) | 0.18 (-0.2 to 0.55) |
| Spain | 1060 (643 to 1695) | 1491 (931 to 2331) | 0.41 (0.28 to 0.57) | 2.73 (1.66 to 4.37) | 3.27 (2.04 to 5.12) | 0.55 (0.43 to 0.68) | 2.49 (1.5 to 4.05) | 2.6 (1.54 to 4.11) | 0.19 (0.04 to 0.35) |
| Sri Lanka | 457 (260 to 773) | 611 (390 to 952) | 0.34 (-0.01 to 0.61) | 2.67 (1.52 to 4.51) | 2.74 (1.75 to 4.28) | 0 (-0.51 to 0.52) | 2.83 (1.6 to 4.68) | 2.57 (1.64 to 4.02) | -0.33 (-0.86 to 0.19) |
| Sudan | 489 (288 to 824) | 847 (510 to 1345) | 0.73 (0.51 to 0.95) | 2.44 (1.44 to 4.12) | 1.95 (1.18 to 3.1) | -0.63 (-0.78 to -0.47) | 2.72 (1.63 to 4.41) | 2.22 (1.37 to 3.51) | -0.58 (-0.71 to -0.45) |
| Suriname | 6 (4 to 10) | 11 (7 to 17) | 0.72 (0.57 to 0.9) | 1.62 (1.01 to 2.63) | 1.87 (1.15 to 2.98) | 0.49 (0.42 to 0.55) | 1.74 (1.11 to 2.79) | 1.82 (1.11 to 2.93) | 0.18 (0.1 to 0.27) |
| Sweden | 309 (187 to 483) | 346 (217 to 535) | 0.12 (0.04 to 0.2) | 3.6 (2.17 to 5.62) | 3.34 (2.1 to 5.16) | -0.23 (-0.41 to -0.05) | 2.91 (1.77 to 4.57) | 2.41 (1.45 to 3.83) | -0.57 (-0.76 to -0.38) |
| Switzerland | 375 (232 to 577) | 417 (260 to 650) | 0.11 (0.02 to 0.21) | 5.46 (3.37 to 8.4) | 4.68 (2.92 to 7.28) | -0.78 (-0.9 to -0.65) | 4.57 (2.8 to 7.25) | 3.45 (2.07 to 5.51) | -1.24 (-1.44 to -1.04) |
| Syrian Arab Republic | 257 (147 to 415) | 387 (242 to 622) | 0.51 (0.3 to 0.87) | 2.02 (1.16 to 3.27) | 2.76 (1.72 to 4.44) | 2.45 (1.81 to 3.09) | 2.28 (1.33 to 3.59) | 2.81 (1.77 to 4.48) | 2.05 (1.46 to 2.64) |
| Taiwan (Province of China) | 501 (301 to 780) | 453 (274 to 702) | -0.09 (-0.2 to 0.03) | 2.46 (1.47 to 3.83) | 1.92 (1.16 to 2.97) | -1.3 (-1.49 to -1.11) | 2.63 (1.6 to 4.04) | 1.41 (0.85 to 2.23) | -2.47 (-2.67 to -2.28) |
| Tajikistan | 159 (93 to 259) | 223 (134 to 357) | 0.4 (0.29 to 0.53) | 2.97 (1.74 to 4.83) | 2.19 (1.31 to 3.52) | -1.8 (-2.19 to -1.41) | 3.23 (1.94 to 5.1) | 2.34 (1.42 to 3.67) | -1.77 (-2.1 to -1.45) |
| Thailand | 1245 (743 to 1960) | 1742 (1090 to 2703) | 0.4 (0.26 to 0.57) | 2.19 (1.31 to 3.45) | 2.61 (1.63 to 4.05) | 0.29 (0.19 to 0.38) | 2.4 (1.46 to 3.71) | 2.14 (1.29 to 3.36) | -0.63 (-0.75 to -0.52) |
| Timor-Leste | 16 (9 to 27) | 20 (13 to 33) | 0.32 (-0.05 to 0.66) | 1.98 (1.14 to 3.42) | 1.46 (0.93 to 2.34) | -1.82 (-2.47 to -1.17) | 2.2 (1.32 to 3.67) | 1.76 (1.12 to 2.76) | -1.34 (-1.85 to -0.81) |
| Togo | 41 (25 to 67) | 93 (56 to 146) | 1.24 (1.07 to 1.45) | 1.13 (0.68 to 1.84) | 1.11 (0.67 to 1.74) | -0.16 (-0.23 to -0.09) | 1.48 (0.9 to 2.3) | 1.43 (0.89 to 2.21) | -0.17 (-0.22 to -0.12) |
| Tokelau | 0 (0 to 0) | 0 (0 to 0) | -0.04 (-0.12 to 0.06) | 1.46 (0.88 to 2.32) | 1.63 (0.99 to 2.55) | 0.39 (0.26 to 0.52) | 1.62 (0.99 to 2.56) | 1.59 (0.96 to 2.5) | -0.09 (-0.16 to -0.01) |
| Tonga | 1 (1 to 2) | 1 (1 to 2) | 0.02 (-0.07 to 0.13) | 1.27 (0.74 to 1.98) | 1.2 (0.73 to 1.89) | -0.12 (-0.33 to 0.08) | 1.53 (0.92 to 2.32) | 1.33 (0.81 to 2.08) | -0.4 (-0.56 to -0.24) |
| Trinidad and Tobago | 21 (13 to 34) | 27 (17 to 43) | 0.29 (0.17 to 0.44) | 1.76 (1.07 to 2.84) | 1.95 (1.2 to 3.12) | 0.54 (0.39 to 0.68) | 1.89 (1.15 to 2.99) | 1.83 (1.12 to 2.97) | 0.11 (-0.02 to 0.24) |
| Tunisia | 212 (128 to 336) | 299 (184 to 466) | 0.41 (0.29 to 0.54) | 2.54 (1.54 to 4.03) | 2.52 (1.55 to 3.94) | -0.01 (-0.07 to 0.05) | 2.76 (1.7 to 4.33) | 2.44 (1.5 to 3.82) | -0.4 (-0.45 to -0.35) |
| Turkmenistan | 103 (60 to 170) | 116 (67 to 191) | 0.12 (0.04 to 0.23) | 2.78 (1.62 to 4.6) | 2.24 (1.31 to 3.69) | -0.72 (-0.82 to -0.61) | 2.91 (1.77 to 4.71) | 2.28 (1.34 to 3.73) | -0.82 (-0.91 to -0.72) |
| Tuvalu | 0 (0 to 0) | 0 (0 to 0) | 0.35 (0.2 to 0.52) | 1.59 (0.95 to 2.51) | 1.65 (0.99 to 2.61) | -0.28 (-0.47 to -0.1) | 1.83 (1.1 to 2.85) | 1.77 (1.06 to 2.79) | -0.49 (-0.66 to -0.32) |
| Uganda | 202 (124 to 330) | 433 (266 to 699) | 1.15 (0.84 to 1.42) | 1.17 (0.72 to 1.91) | 1 (0.61 to 1.61) | -0.6 (-0.73 to -0.48) | 1.44 (0.9 to 2.28) | 1.37 (0.88 to 2.13) | -0.29 (-0.41 to -0.17) |
| Ukraine | 3868 (2326 to 6262) | 2672 (1618 to 4156) | -0.31 (-0.36 to -0.24) | 7.34 (4.41 to 11.88) | 6.2 (3.76 to 9.65) | -1.05 (-1.26 to -0.84) | 6.83 (4.08 to 11.15) | 5.32 (3.22 to 8.47) | -1.28 (-1.46 to -1.09) |
| United Arab Emirates | 55 (33 to 87) | 315 (189 to 487) | 4.73 (4.13 to 5.58) | 2.93 (1.75 to 4.68) | 3.27 (1.96 to 5.06) | 0.44 (0.37 to 0.5) | 3.33 (2.04 to 5.17) | 3.03 (1.82 to 4.7) | -0.35 (-0.37 to -0.34) |
| United Republic of Tanzania | 291 (171 to 474) | 623 (364 to 1002) | 1.14 (0.99 to 1.32) | 1.13 (0.66 to 1.83) | 1.07 (0.62 to 1.71) | -0.18 (-0.22 to -0.15) | 1.38 (0.85 to 2.21) | 1.34 (0.8 to 2.1) | -0.12 (-0.15 to -0.08) |
| United States of America | 6493 (4018 to 10236) | 9990 (6233 to 15481) | 0.54 (0.44 to 0.66) | 2.56 (1.58 to 4.03) | 3 (1.87 to 4.65) | 0.46 (0.25 to 0.68) | 2.33 (1.43 to 3.71) | 2.12 (1.3 to 3.35) | -0.4 (-0.6 to -0.21) |
| United States Virgin Islands | 2 (1 to 3) | 2 (1 to 3) | 0.03 (-0.1 to 0.16) | 1.92 (1.14 to 3.18) | 2.43 (1.52 to 3.68) | 0.55 (0.44 to 0.65) | 2.04 (1.22 to 3.33) | 1.89 (1.14 to 3) | -0.45 (-0.63 to -0.27) |
| Uruguay | 78 (47 to 125) | 89 (55 to 139) | 0.14 (0.04 to 0.26) | 2.47 (1.49 to 3.98) | 2.6 (1.61 to 4.08) | 0.08 (0.04 to 0.11) | 2.36 (1.41 to 3.83) | 2.25 (1.35 to 3.58) | -0.25 (-0.3 to -0.2) |
| Uzbekistan | 614 (360 to 994) | 834 (503 to 1347) | 0.36 (0.26 to 0.48) | 2.93 (1.72 to 4.74) | 2.44 (1.47 to 3.93) | -0.69 (-0.83 to -0.54) | 3.09 (1.87 to 4.9) | 2.51 (1.53 to 4.03) | -0.77 (-0.91 to -0.64) |
| Vanuatu | 2 (1 to 3) | 4 (2 to 6) | 1.18 (0.98 to 1.43) | 1.21 (0.7 to 1.9) | 1.28 (0.76 to 2.04) | 0.06 (-0.23 to 0.34) | 1.53 (0.93 to 2.36) | 1.52 (0.92 to 2.39) | -0.1 (-0.3 to 0.09) |
| Yemen | 297 (177 to 494) | 881 (503 to 1495) | 1.96 (1.43 to 2.91) | 2.18 (1.3 to 3.62) | 2.62 (1.5 to 4.44) | 0.78 (0.52 to 1.04) | 2.68 (1.64 to 4.34) | 2.91 (1.71 to 4.78) | 0.4 (0.21 to 0.58) |
| Zambia | 86 (51 to 144) | 202 (120 to 319) | 1.33 (1.17 to 1.5) | 1.09 (0.64 to 1.81) | 1.03 (0.61 to 1.64) | -0.25 (-0.29 to -0.2) | 1.4 (0.86 to 2.24) | 1.43 (0.86 to 2.2) | 0.03 (-0.03 to 0.08) |
| Zimbabwe | 112 (67 to 182) | 164 (98 to 264) | 0.47 (0.37 to 0.6) | 1.08 (0.65 to 1.76) | 1.05 (0.63 to 1.69) | -0.18 (-0.27 to -0.08) | 1.46 (0.89 to 2.29) | 1.36 (0.85 to 2.13) | -0.28 (-0.35 to -0.2) |

YLDs, years lived with disability; CR, crude rate; ASR, age-standardized rate; EAPC, estimated annual percentage change; UI, uncertainty interval; CI, confidence interval.
